# High estrogen during ovarian stimulation induced loss of maternal imprinted methylation that is essential for placental development via overexpression of TET2 in mouse oocytes

**DOI:** 10.1186/s12964-024-01516-x

**Published:** 2024-02-19

**Authors:** Xueyan Lu, Jiaqin Mao, Chenxi Qian, Hui Lei, Fei Mu, Huijun Sun, Song Yan, Zheng Fang, Jie Lu, Qian Xu, Jie Dong, Danjie Su, Jingjing Wang, Ni Jin, Shuqiang Chen, Xiaohong Wang

**Affiliations:** 1 Reproductive Medicine Center, Department of Gynecology and Obstetrics, Tangdu Hospital, Air Force Medical University, No.1, Xinsi Road, Baqiao District, Xi’an, 710000 Shaanxi Province China; 2 Department of Pharmacy, Xijing Hospital, Air Force Medical University, Xi’an, Shaanxi China

**Keywords:** DNA methylation, Ovarian stimulation, Parthenogenetic activation, Parthenogenetic embryonic stem cells, ERα; TET2, MEST

## Abstract

**Background:**

Ovarian stimulation (OS) during assisted reproductive technology (ART) appears to be an independent factor influencing the risk of low birth weight (LBW). Previous studies identified the association between LBW and placenta deterioration, potentially resulting from disturbed genomic DNA methylation in oocytes caused by OS. However, the mechanisms by which OS leads to aberrant DNA methylation patterns in oocytes remains unclear.

**Methods:**

Mouse oocytes and mouse parthenogenetic embryonic stem cells (pESCs) were used to investigate the roles of OS in oocyte DNA methylation. Global 5-methylcytosine (5mC) and 5-hydroxymethylcytosine (5hmC) levels were evaluated using immunofluorescence or colorimetry. Genome-wide DNA methylation was quantified using an Agilent SureSelectXT mouse Methyl-Seq. The DNA methylation status of mesoderm-specific transcript homologue (*Mest*) promoter region was analyzed using bisulfite sequencing polymerase chain reaction (BSP). The regulatory network between estrogen receptor alpha (ERα, ESR1) and DNA methylation status of *Mest* promoter region was further detected following the knockdown of *ERα* or ten-eleven translocation 2 (*Tet2*).

**Results:**

OS resulted in a significant decrease in global 5mC levels and an increase in global 5hmC levels in oocytes. Further investigation revealed that supraphysiological β-estradiol (E2) during OS induced a notable decrease in DNA 5mC and an increase in 5hmC in both oocytes and pESCs of mice, whereas inhibition of estrogen signaling abolished such induction. Moreover, *Tet2* may be a direct transcriptional target gene of ERα, and through the ERα-TET2 axis, supraphysiological E2 resulted in the reduced global levels of DNA 5mC. Furthermore, we identified that MEST, a maternal imprinted gene essential for placental development, lost its imprinted methylation in parthenogenetic placentas originating from OS, and ERα and TET2 combined together to form a protein complex that may promote *Mest* demethylation.

**Conclusions:**

In this study, a possible mechanism of loss of DNA methylation in oocyte caused by OS was revealed, which may help increase safety and reduce epigenetic abnormalities in ART procedures.

**Supplementary Information:**

The online version contains supplementary material available at 10.1186/s12964-024-01516-x.

## Introduction

Almost 10 million children have been conceived using assisted reproductive technology (ART) globally [1]. However, ART has been associated with an increased risk of adverse gestational and perinatal outcomes, particularly higher rates of low birth weight (LBW) [2, 3]. Ovarian stimulation (OS) plays a vital part in ART, using exogenous gonadotropins to stimulate the ovaries and promote multifollicular development yielding multiple oocytes. The OS appears to be an independent mediator of the risk of LBW [4]. It has also been found that OS results in supraphysiologic levels of maternal serum β-estradiol (E2) on the day of human chorionic gonadotropin (hCG) administration has negative effects on birth weight [5, 6]. One retrospective study indicated that E2 on the day of the hCG trigger was an independent risk factor for LBW [4]. The placenta is a temporary organ that regulates foetal growth and development during gestation. It has been postulated that LBW may be due to deterioration in placenta function, which compromises the transplacental transfer of nutrients to the foetus by ART [7]. Superovulation results in the overgrowth of the placental junctional zone and a lower foetal-to-placental ratio in mouse models [8]. It was also found that OS with the resultant high estrogen levels affect placentation [9]. These results suggest that OS procedures that result in placental deterioration may be responsible for the reduction in birth weight.

The pathogenesis of the increased rate of placental disorders may involve endometrial changes associated with high estradiol levels. In our previous study, we found that OS resulting in aberrant estrogen signaling could lead to disorders in uNK cells, which are crucial for placentation [10]. In addition, the potential effects of high estradiol levels on oocytes during OS must be considered. Our previous studies demonstrated that the peak estradiol concentration is negatively correlated with birthweight in full-term singletons born in vitrified-warmed embryo transfer cycles. These results suggest that the effect on birthweight is primarily due to the influence of high estradiol concentrations on oocytes during OS [11]. This correlation was also supported by another study that demonstrated an inverse association between peak estradiol levels during OS and birth weight of singletons conceived by subsequent frozen transfer [12].

DNA methylation, which is the attachment of a methyl group to a cytosine preceding a guanine base (CpG), is an epigenetic mechanism essential for normal embryonic development [13]. ART procedures influence DNA methylation in oocytes, embryos, and foetuses of humans and mice [13, 14]. Our previous studies found that ART treatment could affect foetal growth by disrupting placental development and function, and suggested that perturbation of DNA methylation of genomic imprinting resulting from embryo manipulation may contribute to these problems in mice [15, 16]. DNA methylation in oocytes is dynamically controlled during oocyte growth, and superovulation has been hypothesized to interfere with this process by altering the timeline of development or affecting the enzymes required for appropriate epigenetic programming [17]. Therefore, it is not surprising that superovulation affected epigenetic regulation in both murine and human studies. In humans, OS can result in methylation errors in imprinted genes in fully grown oocytes [18]. OS in mice results in the loss of maternally imprinted methylation in a dose-dependent manner [19]. In mouse studies, it is also found that OS resulted in a significant decrease in imprinted methylation levels of the early pre-implantation embryos [20–23]. Oocyte-derived DNA methylation plays a major role in trophoblast development and cell adhesion, highlighting its critical and widespread role of oocyte methylation in the development of the placenta [24]. Thus, OS may affect the methylation of genes crucial for placentation in oocytes, resulting in placental deterioration and a reduction in birth weight. However, the mechanism through which OS affects DNA methylation in oocytes remains unclear.

OS results in the loss of maternal DNA methylation in oocytes, which may be due to disruption of the acquisition and/or maintenance of DNA methylation. DNA methylation is controlled by a family of DNA methyltransferases (DNMTs), DNMT1 maintains methylation after DNA replication, whereas DNMT3A and DNMT3B establish DNA methylation patterns de novo [25]. Mammalian 5-methylcytosine (5mC) can still be converted to its unmodified state by the ten-eleven translocation (TET) family of dioxygenases, which includes three members, TET1, TET2, and TET3 [26]. These three TET family members convert 5mC to hydroxymethylcytosine (5hmC) and further oxidize 5hmC to 5-formylcytosine (5fC) and 5-carboxylcytosine (5caC). TET2 lacks N-terminal CXXC-type zinc finger domains and depends on other DNA binding proteins for interaction with DNA [27]. It has also been reported that embryonic imprinting perturbations do not originate from OS induced defects in DNA methylation acquisition, but instead disrupt maternal-effect gene products that are required for the maintenance of methylation during pre-implantation development using BSP [19, 21, 28]. Another study reported that OS resulted in a 50% reduction in global DNA methylation exclusively in the maternal genome of zygotes, as measured by immunofluorescence [29]. We also found that significant genome-wide erasure of CpG methylation from the zygote to the 8-cell stage only occurred in early mouse embryos originating from the OS by whole-genome bisulfite sequencing [20]. The bisulfite sequencing method cannot distinguish between 5mCs and 5hmCs because bisulfite treatment does not convert either modification [30]. 5hmC can be excised by thymine-DNA glycosylase, or passively diluted by DNA replication during cell division, restoring unmodified cytosines [31]. We speculate that OS, resulting in the loss of maternal DNA methylation, may be derived from the convertion of 5mC to 5hmC in oocytes. Therefore, the loss of DNA methylation can be detected in early pre-implantation embryos, but not in oocytes based on bisulfite sequencing.

Supraphysiological level of E2 adversely affect human embryonic stem cell proliferation and histone modifications [32]. E2-treatment has resulted in the rapid loss of global DNA methylation through the upregulation of TET2 in breast cancer cells [33, 34]. TET2 is a transcriptional coactivator of ERα that can regulate the DNA methylation levels on enhancers including estrogen response elements (EREs) [35, 36]. However, it is unclear whether supraphysiological E2 during OS influences the DNA methylation in oocytes. In the present study, we hypothesized that OS resulting in supraphysiological E2 may cause the loss of DNA methylation in oocytes by ERα-TET2 epigenetic axis, which affected maternal DNA methylation that is essential for placental functions and development. Our data revealed that the global decrease in DNA 5mC and the increase in DNA 5hmC in OS mouse oocytes were associated with supraphysiological E2. Moreover, *Tet2* may be a direct transcriptional target gene of ERα, and supraphysiological E2 reduces the levels of 5mC in oocytes via *Tet2*. We have further identified that the maternally imprinted gene *Mest* region is a direct target of the ERα-TET2 complex. This region loses its imprinted methylation under supraphysiological E2 conditions in pESCs. Therefore, this study, elucidates the potential mechanism behind the imprinting in oocytes caused by OS. This understanding may help to enhancing safety and mitigating epigenetic abnormalities associated with ART procedures, ultimately leading to the birth of healthier offspring for patients using ART.

## Methods

### Ethical approval

ICR mice were purchased from Beijing HFK Bio-Technology. Co. Ltd. (Beijing, China). All animal experiments were approved and conducted in accordance with the Institutional Review Board and Experimental Research Ethics Committee of the Tangdu Hospital at the Air Force Medical University.

### Reagents and antibodies

Anti- 5mC (P-1014) and anti-5hmC antibody (P-1018) were obtained from Epigentek (New York, USA). Anti- TET2 (# 36449) and anti-β-actin (# 4967S) antibodies were purchased from Cell Signaling Technology (CST, Massachusetts, USA). Anti-ERα antibody (ab32063) was obtained from Abcam (Massachusetts, UK). Anti-MEST antibody(11118–1-AP) was obtained from Proteintech (Wuhan, China). Si-RNAs for *Esr1* and *Tet2* and respective negative controls were purchased from GenePharma (Suzhou, China). Propidium Iodide (PI) staining buffer (550825) was purchased from BD Biosciences (New York, USA) and DAPI staining buffer (C1002) was obtained from Beyotime (Shanghai, China). MethylFlash Global DNA Hydroxymethylation (5-hmC) Enzyme-Linked Immunosorbnent Assay (ELISA) Easy Kit(P-1032) and MethylFlash Global DNA Methylation (5-mC) ELISA Easy Kit (P-1030) were acquired from Epigentek. Trizol reagent (RRID: AB_2868904) and 5× PrimeScript RT Master Mix (RR036A) were both obtained from Ambion (Texas, USA) and TaKaRa (Shiga, Japan), respectively, while TB Green® Premix Ex Taq™ II (RR420B) was purchased from TaKaRa. TAMXIFEN (10540–29-1) was purchased from MCE (New Jersey, USA).

### Samples

Female ICR mice aged 6–8 weeks of age were selected and randomly divided into two groups: Control and OS. The OS model was generated by intraperitoneal injection of 10 IU of PMSG in PBS followed by 10 IU of hCG 46–48 h later. Immature oocytes at each stage and post-ovulatory cumulus-oocyte complexes (COCs) were isolated by puncturing the ovaries and oviduct ampulla using an insulin syringe in M2 medium after a period of 16 h. Oocytes were assigned to five experimental groups: (1) Control, which consisted of oocytes collected from untreated mice. (2) OS, oocytes were collected from OS mice. (3)1 nM, for which oocytes from the control group were treated with 1 nM E2 for 24 h in vitro. (4)100 nM, in which oocytes from the control group were treated with 100 nM E2 for 24 h in vitro. (5)100 nM + Tamoxifen (TAM), which consisted of oocytes treated with a combination of 100 nM E2 and 10 μM TAM.

### Oocyte collection

Immature oocytes at each stage and post-ovulatory COCs from the Control and OS groups were isolated by puncturing the ovaries and oviduct ampulla with an insulin syringe in M2 medium (Gibco). The cumulus cells of the COCs were removed by repeated pipetting using a fine-bore glass pipette in 0.1% hyaluronidase. Oocytes at various stages were prepared for the subsequent experiments.

### Parthenogenetic activation and developmental rate of embryos

Oocytes with the first polar body were selected for parthenogenetic activation. Mature oocytes were washed thrice in M2 medium and then transferred to 20 mM Sr^2+^ M2 medium for 1 h. Thereafter the oocytes were washed three times with KSOM medium supplemented with 5 μg/mL cytochalasin B and cultured in the same medium for 4 h at 37 °C in 5% CO_2_ atmosphere. Subsequently, 100 presumptive parthenogenetic embryos were cultured in KSOM for subsequent experiments.

### Embryo transplantation

Female mice were mated with ligated male mice on the day of oocyte collection and a plug check was performed the following morning. Female mice with plugs were used as surrogate mothers. Parthenogenetic blastocysts were transferred to the uteri of surrogate mother mice after maturation (10 blastocysts on each side). The gestational sacs were weighed at E10.5, and the placentas of each mouse were mixed as a single mouse sample for Agilent SureSelectXT mouse Methyl-Seq.

### Agilent SureSelectXT mouse methyl-Seq

DNA from the parthenogenetic placentas of the four mice was individually mapped using an Agilent SureSelectXT Methyl-Seq. Genomic DNA was sonicated, treated with bisulfite, and libraries were prepared according to the standard Agilent SureSelectXT Methyl-Seq procedure. The experimental procedure is illustrated in Supplementary Fig. 2. Raw sequencing data were analyzed using the Bismark genome alignment software, samtools tool and the methylkit program in R software. Reference genome and gene annotation information were downloaded from the Ensemble database. Agilent SureSelectXT Methyl-Seq was performed by Shanghai Biotechnology Corporation.

### Isolation of parthenogenetic embryonic stem cells

Parthenogenetic diploid blastocysts were collected and the zona pellucida was digested using Tyrode’s acid. Subsequently, the inner cells and trophoblasts were separated by cutting with a glass needle and transferring the inner cells to 5% KSR-AF medium. After 8–10 days, the flat colonies were cut into small pieces and transferred to a 2% KSR-AF (activin A, bFGF, and 2% KSR in N2B27) medium. The developed colonies were cut and transferred to a 1% KSR-AF (activin A, bFGF, and 1% KSR in N2B27) medium over 10–12 days, and subsequently replaced it with AF medium. The pESCs were derived and self-renewed for 30 passages using accutase at a ratio of 1:3 to 1:5 for 2 days. The composition of the N2B27 medium is presented in the Supplementary Table 1. The AF medium consisted of N2B27 medium supplemented with 20 ng/μL of activin A (R&D, Minnesota, USA) and 12 ng/μL of bFGF (R&D). All using plates were pre-coated by fibronectin (1 mg/mL in PBS; R&D) at least 0.5 h prior to use.

### Alkaline phosphatase staining

The pESCs were seeded on microcarriers, fixed with 4% PFA for 20 min and rinsed three times with PBS. ALP staining of pESCs was performed according to the protocol provided with the ALP Staining Kit (Beyotime). Images were acquired using an inverted optical microscope (Nikon, Oberkochen, Germany).

### Immunofluorescence

Embryonic stem cells were seeded onto a cell culture slide, fully stretched, treated with different E2 levels for 24 h, followed by fixed with 4% paraformaldehyde. After membrane permeabilization and blocking with 0.2% bovine serum albumin (BSA, Beyotime), the cells were incubated with primary antibodies at 4 °C overnight, followed by incubation for 2 h with secondary antibodies at room temperature. For nuclear detection, slides were stained with PI. Fluorescent images of the samples were obtained using a fluorescence microscope (Carl Zeiss, Germany).

For the immunofluorescence analysis of embryos, oocytes or blastocysts were digested using acidic Tyrode’s solution. After fixing with 4% paraformaldehyde, the samples were treated with hydrochloric acid and neutralized using Tris-Base. Slices were permeabilized with Triton X-100, blocked with goat serum, and incubated with the primary antibody overnight at 4 °C. The sections were then incubated with secondary antibodies for 2 h at room temperature. Subsequently, they were counter-stained with PI and sealed using anti-fluorescence quenching sealant.

Immunofluorescent staining was detected using an OLYMPUS laser scanning confocal microscope FV3000. Then ImageJ software (National Institutes of Health, Bethesda, MD, USA) was used to analyze the total fluorescence intensity of a single image based on procedures described elsewhere (Basic Image Analysis and Manipulation in ImageJ/Fiji). Image acquisition for all samples was performed using the same microscope settings and exposure times. The background fluorescence intensity was used as the average intensity in the cytoplasmic region and corrected by subtracting the nuclear or DNA staining intensity. The fluorescence intensity of the embryonic nucleus marker was calculated. The average fluorescence intensity of DAPI in the embryonic nuclei was set to 100%, and this value was compared with the measured nuclear marker intensity to obtain a semiquantitative fluorescence intensity measurement.

### 5mC and 5hmC DNA methylation quantification

Quantification of 5mC and 5hmC DNA methylation was performed using a colorimetric method according to the specifications provided by Epigentek (P-9005). The input DNA (100 ng) was added to bind DNAs to assay wells, which were incubated for 60 min at 37 °C. The binding solution was removed from each well after the 60-min incubation, and the detection complex solution was added to the wells and incubated at room temperature (RT) for 50 min. The colour-developing solution was added (incubation for 30 min), followed by addition of stop solution. Finally, the absorbance was measured at 450 nm using a microplate reader within 2–15 min.

### Bisulfite sequencing polymerase chain reaction (BSP)

Genomic DNA was purified from the parthenogenetic placenta and pESCs and subjected to bisulfite mutagenesis using a DNA Bisulfite Conversion Kit. Bisulfite PCR was performed for the *Mest* or Pleiomorphic Adenoma Gene-Like 1(*Plagl1*) ICR region with two rounds of nested PCR. Primers were designed using the NCBI Primer BLAST tool. Two specific PCR products of 183 bp or 255 bp were obtained after the second round of nested bisulfite PCR. Both specific PCR products were cloned into the pUCm-T vector, and the resultant bacterial colonies were sequenced to determine the methylation status of CpG sites within the region of *Mest* or *Plagl1* ICR. The sequencing results were combined and analyzed using BiQ Analyzer software. The primer sequences are listed in Supplementary Table 2.

### mRNA extraction and quantitative real-time polymerase chain reaction (qRT-PCR)

Total RNA samples from different groups were isolated using the Trizol Reagent. Approximately 1000 ng of each sample was used for cDNA synthesis using the PrimeScript 1st Strand cDNA Synthesis Kit. For oocytes or blastocysts, cDNA products were generated using a REPLI-g WTA Single Cell Kit according to the manufacturer’s protocol. The cDNA products were diluted 10 times for qRT-PCR, which was performed on a CFX96 Real-Time System using the TB Green Premix Ex Taq II. The relative expression of target genes was calculated using the ΔCt method (2 ^–ΔΔCt^), with actin as an internal control. The primer sequences used are listed in Supplementary Table 3.

### Western blot analysis

The parthenogenetic placenta and pESCs were lysed using radioimmunoprecipitation (RIPA) lysis buffer containing protease and phosphatase inhibitors. Proteins were separated on 4–20% precast protein gels and transferred onto PVDF membranes. The membranes were blocked with 5% skim milk for 2 h at room temperature and incubated with primary antibodies overnight. After washing with TBST, the membranes were incubated with horseradish peroxidase-labelled secondary antibodies for 1 h at room temperature, followed by enhanced chemiluminescence detection.

### Small interfering RNA transfection

Cells were seeded in 6-well plates (3 × 105 cells/ well) and cultured in complete medium. Cells were transfected at 50% confluence with negative control siRNA, *Esr1*-siRNA and *Tet2*-siRNA using X-tremeGENE siRNA transfection reagent (Roche, Basel, Switzerland) according to the manufacturer’s instructions; the sequences of siRNAs are shown in Supplementary Table 4. After transfection for 48–72 h, the cells were collected for subsequent experiments.

### Chromatin immunoprecipitation (ChIP) assay

A chromatin immunoprecipitation (ChIP) assay followed by qPCR (ChIP-qPCR), was performed according to the manufacturer’s protocol (CST#56383S). Briefly, to cross-link the proteins to DNA, an appropriate amount of 37% formaldehyde solution was added to the medium to achieve a final concentration of 1% for 10 min at room temperature. Glycine solution was added to terminate the fixation reaction. After cell and nuclear lysis, the DNA in the cell lysates was fragmented into less than 1 kb chromatin fragments using an ultrasonic apparatus. Next, the protein-bound chromatin was immunoprecipitated with the ERα antibody and eluted from protein G magnetic beads. DNA fragments were purified using CST DNA purification columns, and further CHIP analysis was performed using qRT-PCR. The primer sequences are listed in Supplementary Table 5. Percent input of each ChIP sample was calculated as follows: % Input = 2^ (Ct Input−Ct ChIP) × 100.

### Co-inmunoprecipitation (Co-IP)

Harvested pESCs were lysed with IP lysis buffer on ice for 15 min, and the mixture was thoroughly mixed once every 5 min. Cell debris was removed by centrifugation at ~ 13,000×g for 10 min and the supernatant was transferred to a new tube for protein concentration determination and Immune Complex analysis. We combined 1000 μg of cell lysate with 2.5 μg of ERα/IgG antibody per sample in a microcentrifuge tube and diluted to 500 μL by adding IP Lysis Buffer. Followed by incubation overnight at 4 °C to form an immune complex. Next, we added 25 μL of Pierce Protein A/G Magnetic Beads to the immune complex to mix on 4 °C overnight. The immunocomplexes were washed two times with the lysis buffer and one time with ultrapure water, boiled at 100 °C for 10 min with 4x Laemmli protein sample buffer (Bio-rad, California, USA) and analyzed by western blot.

### Statistical analysis

Data were expressed as the means ± standard deviation (SD) and analyzed using the SPSS 21.0 statistic software program. For comparisons between two groups, we used an unpaired two-tailed t-test. One-way analysis of variance (ANOVA) multiple comparison test was performed among the three groups. Statistical significance was set at *P* < 0.05.

## Results

### OS altered the levels of global DNA 5mC and 5hmC in GV and MII oocyte in mice

**Fig. 1:**
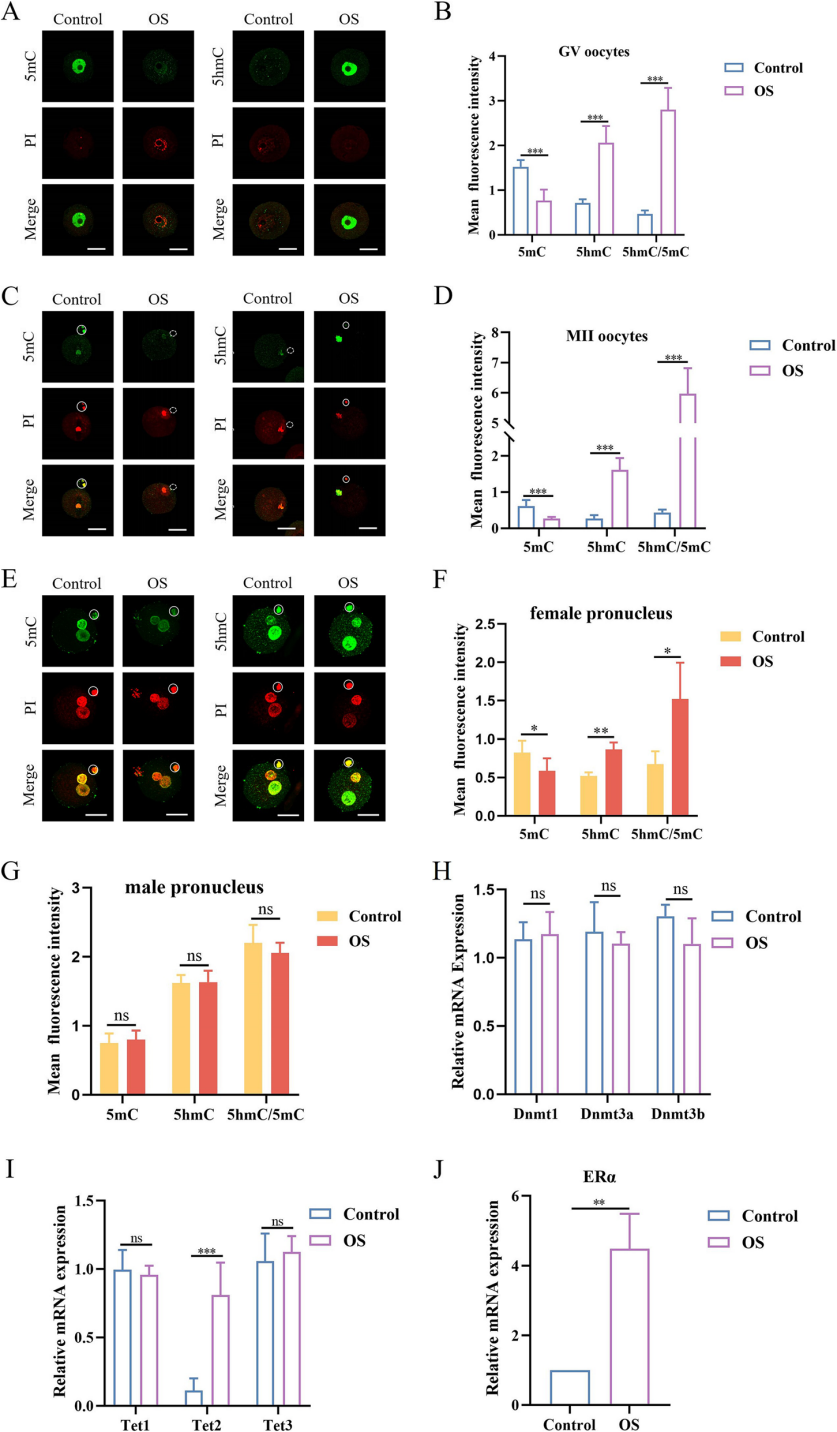
Effect of ovarian stimulation (OS) on relative intensity of 5-methylcytosine (5mC) and 5-hydroxymethylcytosine (5hmC) fluorescence of oocytes and pronuclear-stage embryos. **A**, **C** Representative immunofluorescence images of 5mC and 5hmC in germinal vesicle (GV) and metaphase II (MII) oocytes, red fluorescence with Propidium Iodide (PI) staining and green fluorescence with Dylight 488 staining, white circle indicates the first polar body. **B**, **D** Quantification of average fluorescent intensities with 5mC and 5hmC staining within each group (*n* = 30). **E** Representative immunofluorescence images of 5mC and 5hmC in pronuclear-stage embryos, white circle indicates the second polar body. **F**, **G** Quantification of average fluorescent intensities with 5mC and 5hmC staining of female and male pronucleus (*n* = 20). **H** Relative mRNA level of Analysis of RNA levels for DNA methyltransferase 1, 3a and 3b (*Dnmt1*, *Dnmt3a*, *Dnmt3b*, *n* = 8). **I**-**J** Expression level of ten-eleven translocation 1,2 and 3 (*Tet1*, *Tet2*, *Tet3*) and estrogen receptor alpha (*ERα*) within each group (*n* = 8). Data are expressed as the means ± standard deviation (SD), ns: Not Statistically Significant, *P < 0.05, **P < 0.01, ***P < 0.001(unpaired two-tailed t test). Scale bar = 30 μm

We analyzed whether OS could lead to an increase in estrogen levels. We tested the serum E2 levels of mice in three groups: (1) control group 1, non-pregnant mice; (2) control group 2, pregnant mice after mating with male mice. Serum was collected at 10.5d post-coitus; and (3) experimental group, OS mice, serum was collected 12 h after hCG injection. The OS group showed higher serum E2 levels than the two control groups (Supplementary Fig. 1). Therefore, OS results in supraphysiological E2 in mice. Next, we examined the effect of OS on DNA 5mC and 5-hmC levels using immunofluorescence in germinal vesicle (GV) and metaphase II (MII) oocytes (Fig. 1A-D). Global DNA methylation levels were remarkably decreased, and DNA 5-hmC levels were significantly increased in OS GV oocytes compared to control oocytes (Fig. 1B). The same trend was observed in MII oocytes (Fig. 1D), suggesting that DNA demethylation occurred in oocytes obtained by OS. We also explored the effect of OS on DNA 5mC and 5hmC in female and male pronucleus zygotes under normal fertilization conditions (Fig. 1E-G). Interestingly, after fertilization, compared to the control zygote, the OS female pronucleus showed a significant decrease in 5mC and an elevation in 5hmC (Fig. 1F). However, there were no differences in 5mC and 5hmC levels in male pronucleus between the control and OS groups (Fig. 1G). Prior research indicates that in the zygote, the paternal genome undergoes active demethylation, whereas the maternal genome is subject to passive demethylation through DNA replication during the cleavage stage. Active demethylation of the male pronucleus in mouse zygotes is mediated by Tet3 [37–39]. This could explain why an increase in TET2 levels in the oocyte-sperm (OS) zygote does not influence DNA methylation within the male pronucleus. We then explored the effect of OS on the expression of the three DNMTs and TET family members. These include DNMT1, DNMT3A, DNMT3B, TET1, TET2 and TET3. We found no statistically significant differences in the expression of *Dnmt1*, *Dnmt3a*, or *Dnmt3b* between the two groups (Fig. 1H). The expression of *Tet2* was significantly increased in the OS group but not *Tet1* and *Tet3* (Fig. 1I). Thus, we speculate that OS results in the loss of maternal DNA methylation which may be due to the conversion of 5mC to 5hmC in oocytes by the upregulation of *Tet2*. It has been reported that estrogen signaling promotes TET2 expression via ERα [34]. As shown in qRT-PCR, the expression of *ERα* was significantly increased in the oocytes obtained by OS (Fig. 1J). These findings indicate that OS induced DNA demethylation in oocytes may occur via estrogen signaling.

### Global DNA 5mC and 5hmC levels are affected by OS in the parthenogenetic pre-implantation embryos

**Fig. 2:**
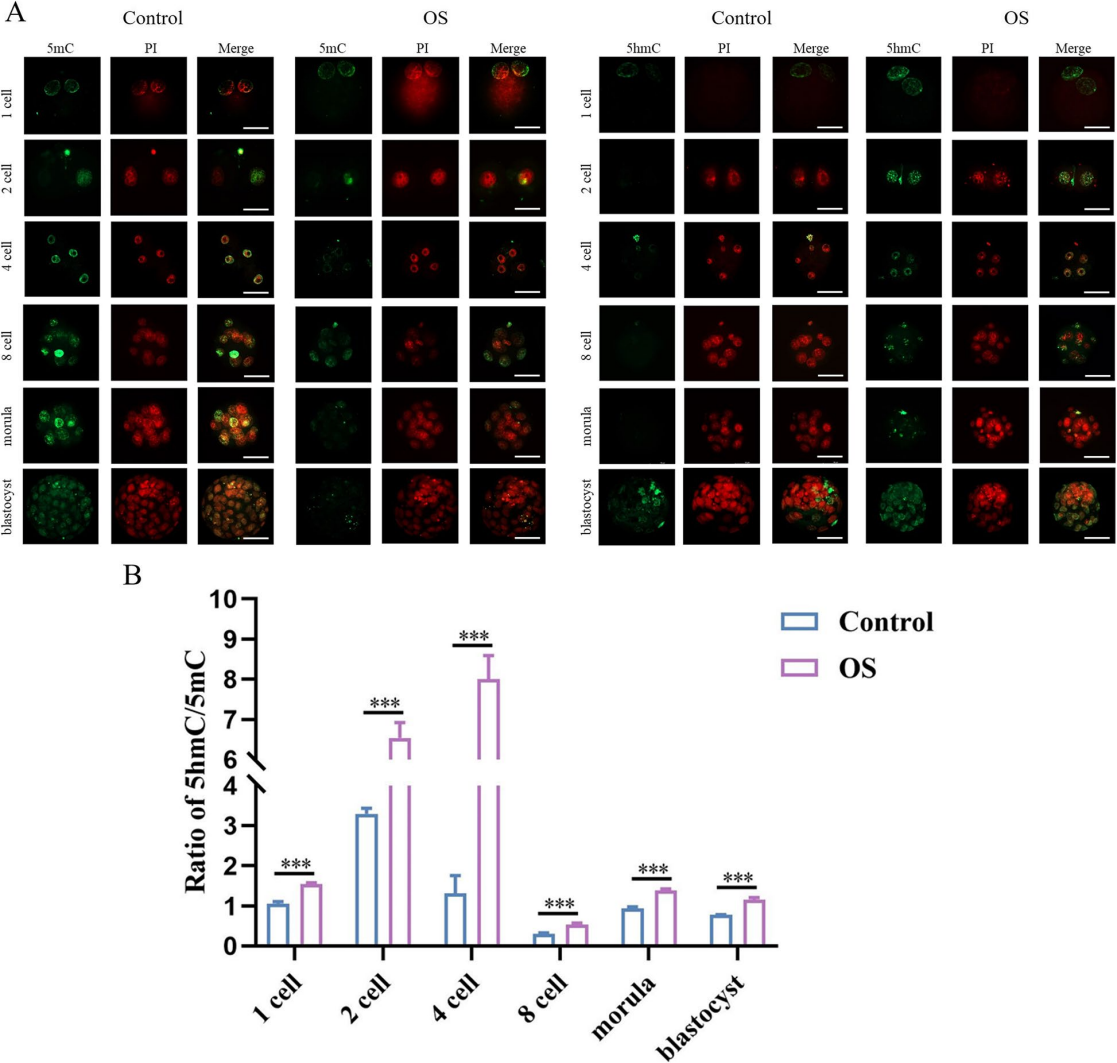
Global DNA 5-methylcytosine (5mC) and 5-hydroxymethylcytosine (5hmC) levels are affected by ovarian stimulation (OS) in the parthenogenetic early embryos. **A** Representative developmental images of parthenogenetically activated embryos with each stage. **B** Analysis of ratios of 5mC/5hmC within each group (*n* = 28–60). Data are expressed as the means ± standard deviation (SD), ***P < 0.001. (unpaired two-tailed t test). Scale bar = 100 μm

Parthenogenetic embryos, formed by the activation and diploidization of oocytes, do not contain paternal genomes. We then used parthenogenetic embryos to further analyze whether OS DNA hypomethylation of DNA is maintained in pre-implantation embryos and placentas. The levels of 5mC and 5hmC in the 1-cell, 2-cell, 4-cell, 8-cell, morula and blastocyst stages were analyzed using whole-mount immunofluorescence staining (Fig. 2A). We found that global DNA 5mC levels were significantly lower and 5hmC levels were significantly higher in parthenogenetic embryos at each stage in the OS group (Fig. 2B). These results suggested that OS-induced hypomethylation of DNA in oocytes was maintained in pre-implantation embryos.

### The DNA methylation level of *Mest* was significantly decreased in the parthenogenetic placentas originating from OS

**Fig. 3:**
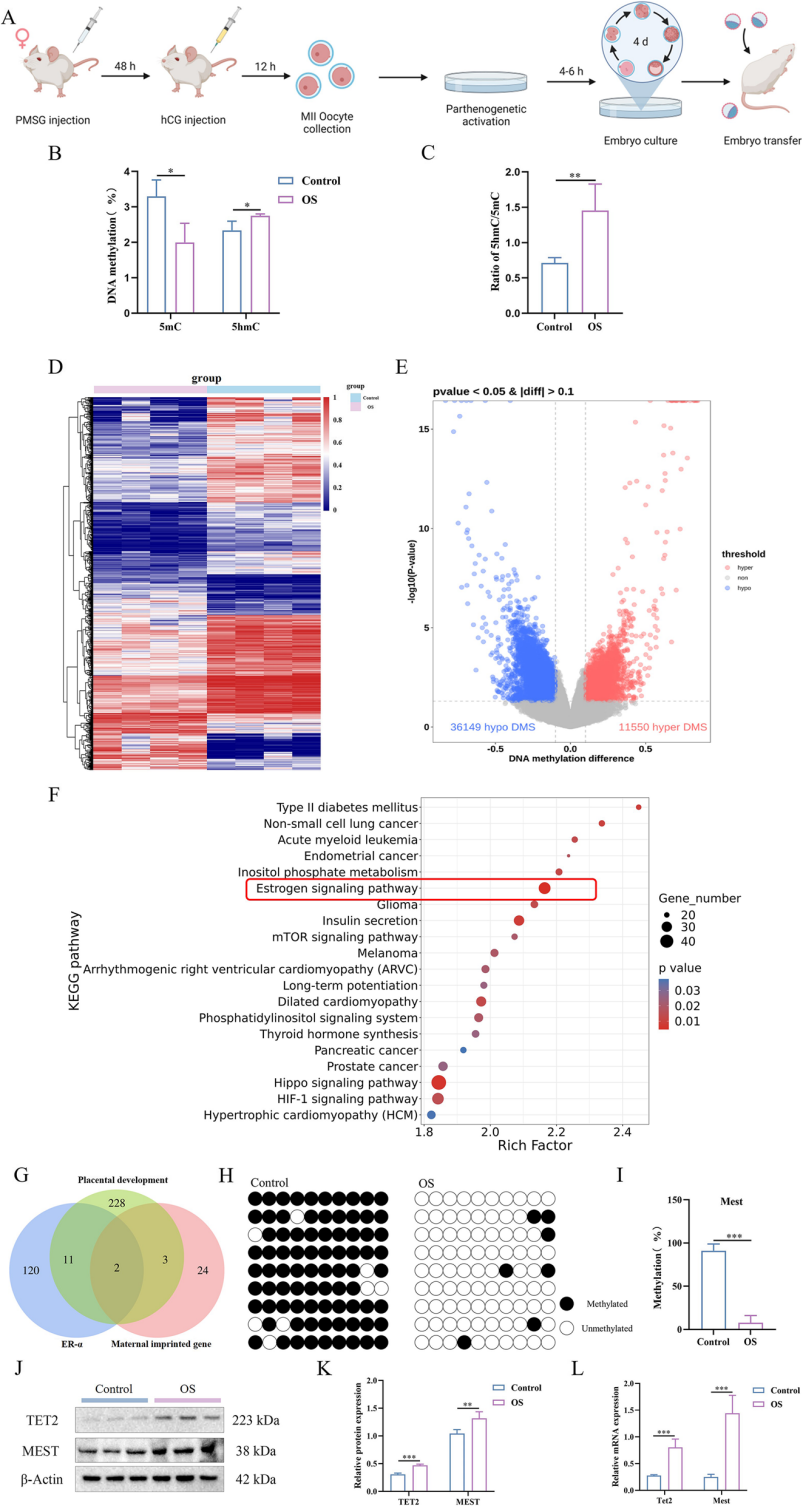
Association between ovarian stimulation (OS) and DNA methylation of parthenogenetic placenta. **A** Flow diagram illustrating the parthenogenetic activation of oocytes, their development into blastocysts, and the subsequent transfer to pseudopregnant female mice. **B**, **C** The percentage of methylated DNA, hydroxymethylated DNA and the 5-hydroxymethylcytosine (5hmC)/5-methylcytosine (5mC) ratio were measured using an Enzyme-Linked Immunosorbent Assay (ELISA) kit (*n* = 4). **D** Cluster heat map of the 1000 most differentially methylated cytosine preceding a guanine base (CpG) sites in placenta tissue DNA samples from 4 mice. Each row represents differentially methylated CpGs (DMCs), and each column represents a sample. **E** DMCs distribution of OS and Control groups depicted in a volcano plot. Non-significant CpGs are shown in grey color, while hypermethylation and hypomethylation CpGs are shown in red and blue, respectively. **F** Top 20 KEGG enrichment results of DMCs annotated genes. **G** The Venn diagram analysis was used to determine the interaction of hypermethylated maternal imprinted genes, placenta-developed genes and transcription factors targeted by estrogen receptor alpha (ERα). **H**, **I** Analysis of methylation levels at CpG sites within the differentially methylated region (DMR) in the mesoderm-specific transcript homologue (*Mest*) promoter, evaluated by bisulfite sequencing PCR (BSP, *n* = 9). Each row of eight CpG sites within a group represents a single bisulfite-treated clone with methylated CpG (●) or unmethylated CpG (○). **J**, **K** Protein expression of ten-eleven translocation 2 (TET2) and MEST was determined using western blot analysis (*n* = 3). β-Actin was used as an internal control to quantify protein amounts. **L** Analysis of RNA levels for *Tet2* and *Mest* was performed within each group (*n* = 4). Data are expressed as the means ± standard deviation (SD), *P < 0.05, **P < 0.01, ***P < 0.001(unpaired two-tailed t test)

To study placental DNA methylation levels after implantation, parthenogenetic blastocysts from the control and OS groups were transferred to pseudopregnant females on postcoital day 3.5 (Fig. 3A). Parthenogenetic placentas were harvested on gestational day (gd) 10.5. Compared to control placentas, the global DNA methylation level was lower in placentas from the OS group (Fig. 3B). Furthermore, increased levels of global DNA 5-hmC and 5-hmC/5mC ratios were observed in parthenogenetic placentas originating from the OS (Fig. 3B, C). In addition, in placentas with normal fertilization at 10.5d of pregnancy, we found that 5mC was significantly decreased in 5mC and increased in 5hmC in the OS group compared with the control group (Supplementary Fig. 1B, 1C). These results suggest that the OS induced hypomethylation of DNA in oocytes is maintained in mid-gestation placentas.

Next, we sought to identify regions that exhibited methylation differences between parthenogenetic placentas from the control and OS groups using Agilent SureSelectXT mouse Methyl-Seq (Supplementary Fig. 2). In total, we identified 47,699 different methylated CpGs (DMCs) between the two groups, among which 36,149 DMCs were hypomethylated and 11,550 DMCs were hypermethylated in the OS placentas compared to the control (Fig. 3D, E). These results are consistent with the global methylation results, suggesting lower DNA methylation levels in OS placentas. To fully understand the impact on regions that gained or lost methylation in the placenta, KEGG enrichment analysis was performed. Interestingly, gene changes in DNA methylation were mainly involved in estrogen signaling, metabolism, mTOR, and other pathways that are important for placental function (Fig. 3F). As OS resulted in increased expression of ERα, induced DNA demethylation and imprinted genes demonstrated to play important functions prenatally regulating placental development. By cross-linking the ERα regulated genes, placental development related genes and maternal imprinted genes within the 36,149 hypomethylated methylated sites, we found that two maternal imprinted genes, *Mest* and *Plagl1* exist in the three regions simultaneously (Fig. 3G). To verify the methylation levels of *Mest* and *Plagl1*, the methylation status was detected using BSP. Compared with the control placentas, the DNA methylation level of *Mest* was significantly decreased in the placentas of the OS group (Fig. 3H, I), but the DNA methylation level of *Plagl1* was not different between the two groups (Supplementary Fig. 3A, 3B). Western blot analysis showed that TET2 and MEST levels were increased in the OS group (Fig. 3J-K). Moreover, OS resulted in increased RNA expression levels of *Tet2* and *Mest* in parthenogenetic placentas compared to those in the control group (Fig. 3I).

### Supraphysiological E2 reduces the global levels of DNA 5mC in GV and MII oocyte by upregulation of TET2 in mice

**Fig. 4:**
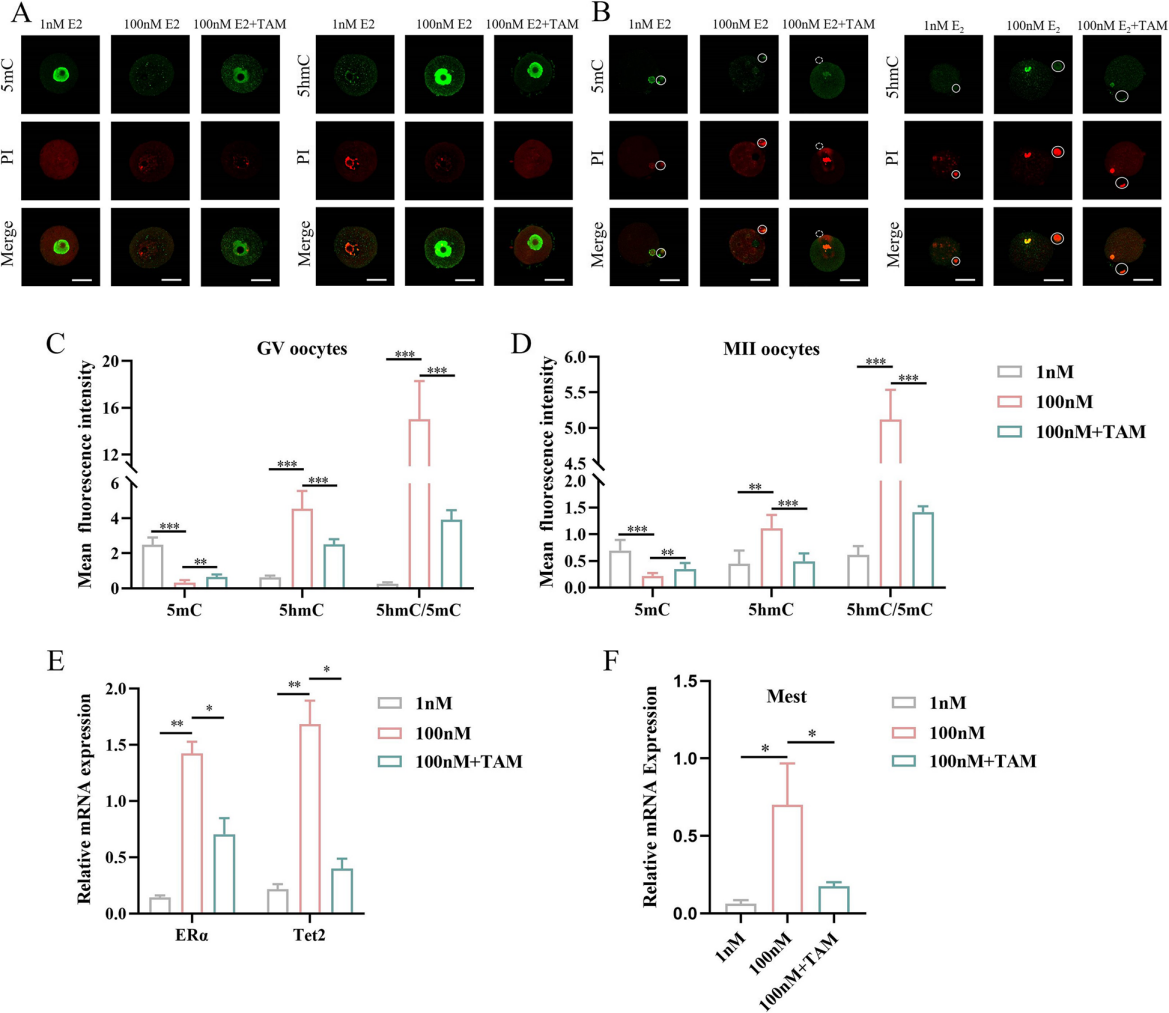
Effect of β- estradiol (E2) on oocytes DNA methylation and associated proteins. **A**, **B** Representative immunofluorescence images of 5-methylcytosine (5mC) and 5-hydroxymethylcytosine (5hmC) in germinal vesicle (GV) and metaphase II (MII) oocytes, showing red fluorescence with Propidium Iodide (PI) staining and green fluorescence with Dylight 488 staining, white circle indicates the first polar body. **C**, **D** Quantification of average fluorescent intensities with 5mC and 5hmC staining in each group (*n* = 30). **E**, **F** Analysis of RNA levels for ten-eleven translocation 2 (*Tet2*, *n* = 8), estrogen receptor alpha (*ERα*, *n* = 8) and mesoderm-specific transcript homologue (*Mest*, *n* = 8) in each group. Data are expressed as the means ± standard deviation (SD), *P < 0.05, **P < 0.01, ***P < 0.001 (One-way ANOVA multiple comparisons test). Scale bar = 30 μm

As E2-treatment resulted in the rapid loss of global DNA methylation through the upregulation of TET2 in breast cancer cell [36, 40]. OS induces supraphysiological E2 production in both humans and mice. We tested whether supraphysiological E2 production caused by OS affects the DNA methylation in oocytes. E2 levels of 1 nM have been found to approximate the physiological concentrations in women in the mid-secretory phase, whereas levels of 100 nM are characteristic of supraphysiological concentrations in women undergoing controlled ovarian hyperstimulation [41, 42]. Thus, we opted to use experimental concentrations of 1 nM and 100 nM. To further investigate whether supraphysiological E2 affects DNA methylation in oocytes, immature oocytes from the ovaries were isolated and cultured for 24 h in vitro under 1 nM or 100 nM E2 for 24 h according to previous literatures [43]. As expected, the level of 5mC in GV oocytes treated with 100 nM E2 significantly decreased, but 5hmC levels were higher than those cultured in 1 nM E2 (Fig. 4A). Accordingly, for MII oocytes, E2 treatment resulted in a steady decrease in DNA methylation levels and an increase in 5hmC levels over a 24-hour period (Fig. 4B). However, after adding TAM (ERα inhibitors) to the IVM medium with100 nM E2, the DNA demethylation was significantly reversed (Fig. 4C, D). Furthermore, the expression of *ERα, Tet2* and *Mest* was increased significantly after exposure to 100 nM E2 than in 1 nM (Fig. 4E, F). It was also found that the expression of *Mest* was significantly increased in OS oocytes compared to that in the control (Supplementary Fig. 4). However, after adding TAM to the IVM medium with 100 nM E2, the expression of *ERα*, *Tet2* and *Mest* was significantly reversed. This finding suggests that supraphysiological E2 can affect DNA methylation by modulating *Tet2* expression, which may participate in the OS induced loss of DNA methylation in oocytes.

### Supraphysiological E2 induces the expression of TET2 and alters the DNA methylation level of *Mest* in mouse parthenogenetic embryonic stem cells

**Fig. 5:**
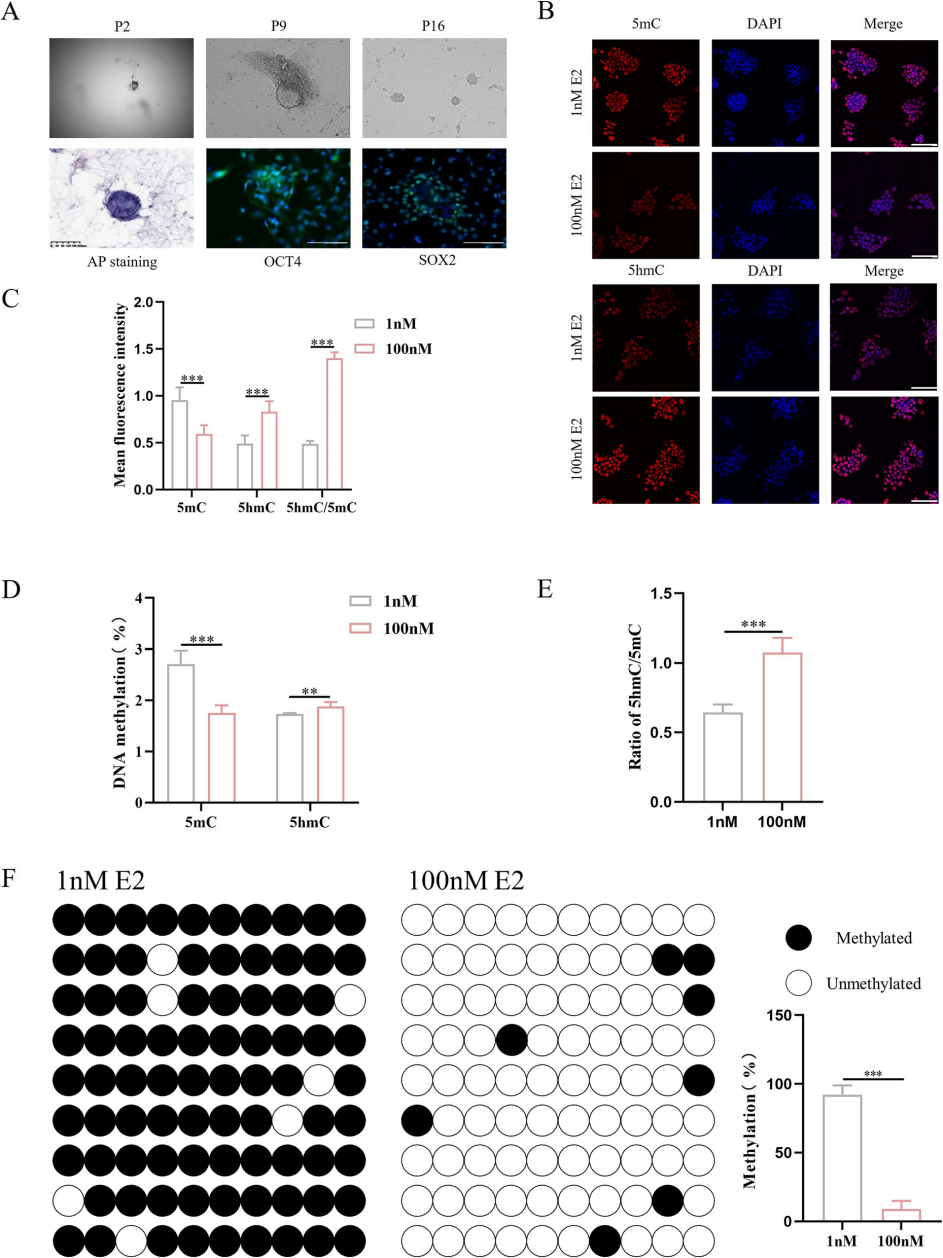
Supraphysiological β-estradiol (E2) induces the expression of ten-eleven translocation 2 (TET2) and alters the levels of global DNA 5-methylcytosine (5mC) and 5-hydroxymethylcytosine (5hmC) in mouse parthenogenetic embryonic stem cells (pESCs). **A** The derivation process and AP staining of pESCs; P2: passage 2; P9: passage 9; P16: passage 16. Representative image of alkaline phosphatase (ALP) staining in pESCs, Octamer-Binding Transcription Factor 4 (OCT4) and sex-determining region Y-box 2 (SOX2) expression in pESCs-blue fluorescence with 4,6-diamino-2-phenyl indole (DAPI) nuclear staining and green fluorescence with Dylight 488 staining. **B** Representative immunofluorescence images of pESCs as shown with 5mC and 5hmC (red fluorescent) and nuclear DAPI staining (blue fluorescence) within each group. **C** Quantification of average fluorescent intensities for 5mC and 5hmC staining within each group (*n* = 6). **D**, **E** The percentage of methylated DNA, hydroxymethylated DNA and 5-hmC/5mC ratio were detected by Enzyme-Linked Immunosorbent Assay (ELISA) kit (*n* = 6). **F** Analysis of methylation levels at cytosine preceding a guanine base (CpG) sites within the differentially methylated region (DMR) in the mesoderm-specific transcript homologue (Mest) promoter, evaluated using bisulfite sequencing PCR (BSP, *n* = 9). Data are expressed as the means ± standard deviation (SD), **P < 0.01, ***P < 0.001. Scale bar = 100 μm (unpaired two-tailed t test)

Parthenogenetic embryonic stem cells exhibit imprinting consistent with an exclusively maternal lineage, that shows a typical bimaternal imprinting pattern [40, 44]. Therefore, we used pESCs as an valuable in vitro model system to study the molecular mechanisms of supraphysiological E2 in oocytes DNA methylation. PESCs were generated from parthenogenetic blastocysts. After 30 passages, the pESCs formed saucer-shaped colonies and showed positive staining of alkaline phosphatase (Fig. 5A).

Furthermore, we performed immunofluorescence and found that pESCs expressed the main pluripotency marker, octamer-binding transcription factor 4 (OCT4), and sex-determining region Y-box 2 (SOX2) (Fig. 5A). To further test whether supraphysiological E2 leads to DNA demethylation, pESCs were exposed to 1 nM or 100 nM E2. When treated with 100 nM E2, there was a decrease in 5mC and an increased in 5hmC compared to 1 nM E2, as shown by whole-mount immunofluorescence staining (Fig. 5B, C). Consistent with the immunofluorescence results, colorimetric analysis revealed that high estrogen levels induced the downregulation of DNA methylation levels and an increase in hydroxylation levels in parthenogenetic stem cells (Fig. 5D, E). In addition, 100 nM E2 caused a loss of DNA methylation of the *Mest* gene compared to 1 nM E2 (Fig. 5F). Together, these results demonstrate that high estrogen may affect DNA methylation through ERα-TET2 axle in pESCs.

### ERα recruits TET2 to activate *Mest* demethylation in mouse parthenogenetic embryonic stem cells under supraphysiological E2

**Fig. 6:**
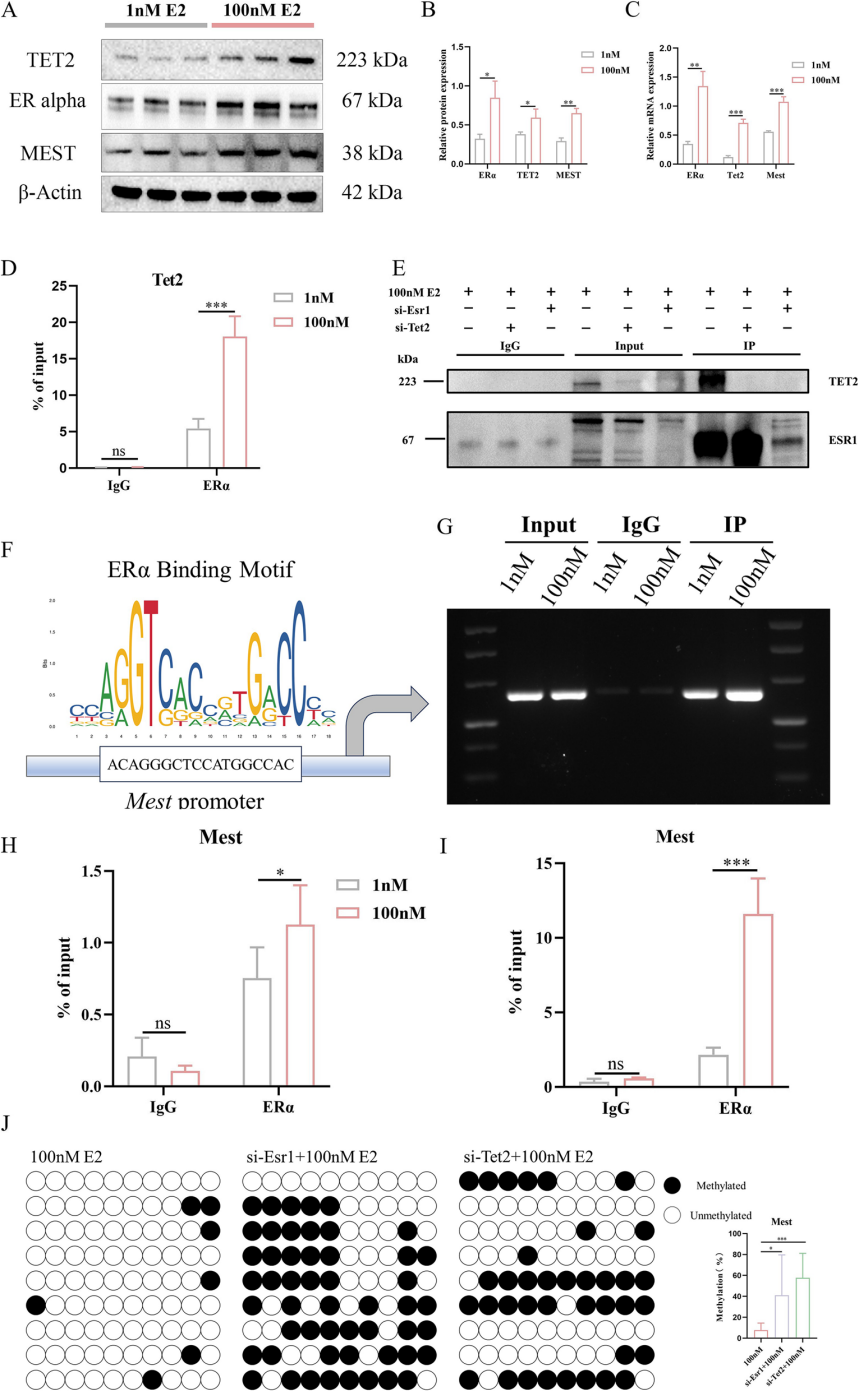
Estrogen receptor alpha (ERα) co-binds to ten-eleven translocation 2 (TET2) and regulates the levels of mesoderm-specific transcript homologue (Mest) imprinted methylation in mouse parthenogenetic embryonic stem cells (pESCs). **A**, **B** Protein expression of TET2 and ERα following 24 h of pESCs culture with 1 nM or 100 nM was determined using western blot analysis (*n* = 6). **C** Quantification of Tet2, ERα and Mest expression levels within each group by quantitative real-time polymerase chain reaction (qRT-PCR, *n* = 6). **D**, **E** Chromatin immunoprecipitation (ChIP) and Co-immunoprecipitation (CoIP) assay using ERα as bait protein demonstrated the interaction between ERα and TET2 (*n* = 3). Anti-rabbit IgG immunoprecipitation (IP) was used as a negative control. **F** Jaspar online website was used to predict binding sites of ERα to Mest. **G**, **H** QRT-PCR was performed with the indicated primer pair using an anti-ERα antibody immunoprecipitated DNA fragment as template (*n* = 6). **I** PCR products were separated by agarose gel electrophoresis and subjected to quantitative analysis (*n* = 3). **J** Analysis of methylation levels at cytosine preceding a guanine base (CpG) sites within the differentially methylated region (DMR) in the Mest promoter were performed using bisulfite sequencing PCR (BSP, *n* = 9). Data are expressed as the means ± standard deviation (SD), *P < 0.05, **P < 0.01, ***P < 0.001. (Unpaired two-tailed t test was used to compare differences among two groups and one-way ANOVA multiple comparisons test was performed among the three groups)

It was demonstrated that TET2 is a direct target of ERα in breast cancer cells [35, 36]. Next, we tested whether high estrogen treatment affects the expression of TET2 in pESCs. We found that the protein and RNA expression levels of TET2, ERα and MEST were significantly upregulated under 100 nM E2 compared with 1 nM E2 (Fig. 6A-C). In addition, our ChIP-qPCR analysis showed that ERα was recruited to the upstream regulatory regions of the *Tet2* gene upon E2 treatment in pESCs (Fig. 6D). These results suggested *Tet2* may be a direct target of ERα in pESCs.

TET2 is a component of the estrogen receptor complex in multiple breast cancer models [35]. Subsequently, we used an anti- ERα antibody as bait for Co-IP assay, and observed a distinct immunoblotting of TET2, which confirmed that ERα and TET2 combine to form a protein complex in pESCs (Fig. 6E). TET2 co-binds with ERα at enhancer elements of ERα target gene to regulate DNA modification dynamics at these regulatory elements. As supraphysiological E2 can affect *Mest* DNA methylation in pESCs, we speculated that MEST may be a direct target of ERα. ChIP-PCR analysis was conducted to evaluate whether ERα can effectively target the binding motifs in *Mest*. We also evaluated whether the ERα can effectively target the binding motifs in *Plagl1* but no evidence of binding was found (Supplementary Fig. 3C). The results confirmed that ERα preferentially bound to the *Mest* promoter in pESCs (Fig. 6G, H). ChIP-qPCR analyzes also revealed that ERα binds to the upstream regulatory regions of the *Mest* gene (Fig. 6I). The results indicated that ERα may regulate *Mest* methylation in pESCs via TET2 under high E2 condition. Then, we used a specific siRNA to knock down endogenous *Esr1* or *Tet2* expression in pESCs. We chose si*Esr1*–1 and si*Tet2*–4 for the follow-up experiments as they were the most efficient siRNAs (Supplementary Fig. 5) and analyzed the methylation levels at CpG sites within the differentially methylated region (DMR) in the *Mest* promoter. The results showed that knocking down the *ERα* expression in pESCs could rescue the loss methylation of *Mest* caused by supraphysiological E2. Similar results were obtained by knocking down *Tet2* expression in pESCs (Fig. 6J).

However, the rescue process remains incomplete, and various factors could account for this. Firstly, our rescue involved siRNA knockdown of *Esr1* or *Tet2*, but the knockdown may not have been completed, leading to low expression level of *ERα* and *Tet2*. Another study has found that estrogen can promote DNA demethylation through ERβ, although the mechanism is unclear [45]. Therefore, other pathways may be involved in the demethylation of *Mest* induced by high estrogen level.

## Discussion

**Fig. 7:**
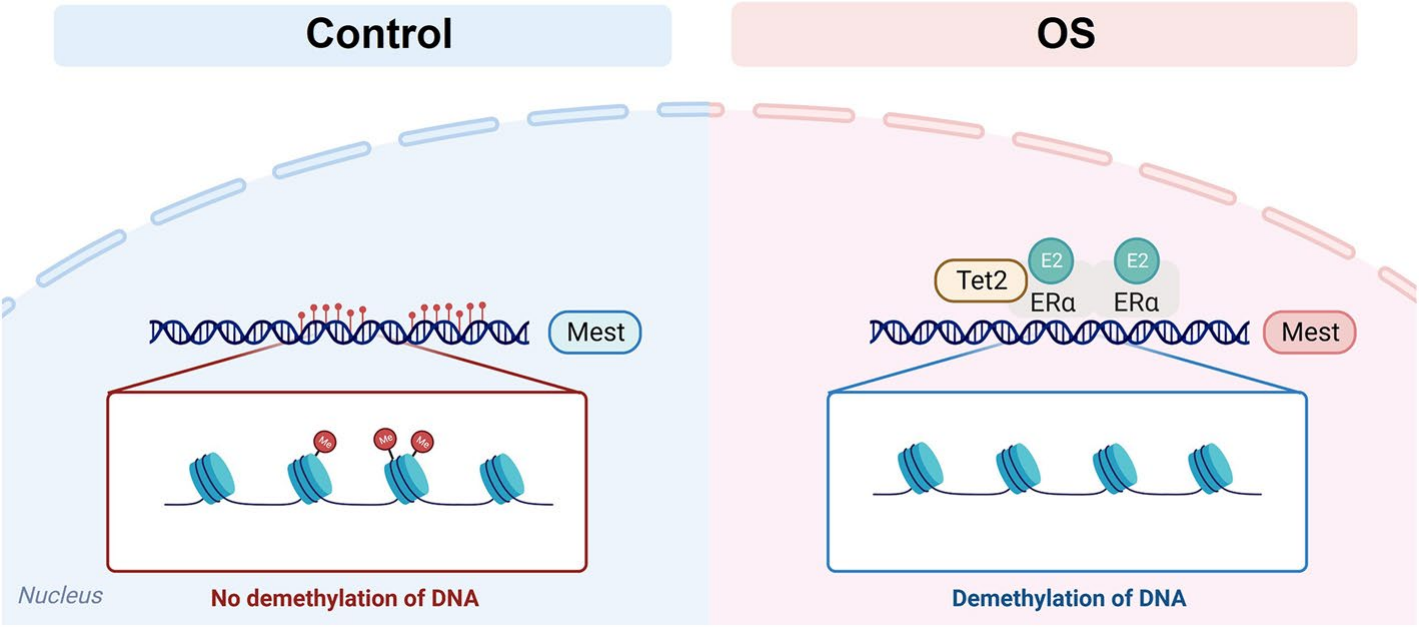
The schematic diagram of the aberrant DNA methylation patterns induced by ovarian stimulation

ART has been associated with a range of adverse obstetric and perinatal outcomes, especially, particularly with low birth weight. Aberrant methylation of imprinted genes in both gametes and early embryos resulting from ART may contribute to these adverse outcomes [46, 47]. As an essential component of assisted reproductive technology, OS disrupt DNA methylation during gametogenesis and early embryogenesis. Studies revealed that OS plays a causal role in the dysregulation of DNA methylation in humans and mice [18, 19]. However, the mechanism by which aberrant DNA methylation patterns are caused by OS remains unclear. Our data revealed that the global decrease in DNA 5mC and the increase in 5hmC in mouse oocytes may have been caused by supraphysiological E2 during OS. Moreover, supraphysiological E2 induced a significant decrease in DNA 5mC and an increase in 5hmC in oocytes, female pronucleus of normal fertilization zygotes, and mouse pESCs, whereas inhibition of estrogen signaling or TET2 depletion abolished this induction. Furthermore, *Tet2* may be a direct transcriptional target gene of ERα, and TET2 co-binds with ERα to induce DNA demethylation of the ERα target genes under supraphysiological E2 conditions (Fig. 7). To the best of our knowledge, this is the first study to reveal a possible mechanism underlying the loss of imprinting in oocytes and early embryos caused by OS.

Studies at the mouse zygote stage have detected a reduction in global DNA methylation in maternal pronuclei associated with OS [23]. To investigate the possible impact of OS on DNA methylation reprogramming during oogenesis, we performed immunofluorescence staining of GV and MII stage oocytes using antibodies against 5mC and 5hmC. The results of our whole-mount immunofluorescence staining indicated a negative effect of OS on DNA methylation levels in GV and MII oocytes. Interestingly, in the control zygote, the OS female pronucleus showed a significant decrease in 5mC and an elevation in 5hmC but not in the male pronucleus. Subsequent RT-qPCR analyzes of the *Tet1*, *Tet2*, and *Tet3* genes revealed that OS significantly induced the expression of *Tet2*, but no change was observed in the expression of *Tet1* and *Tet3*. The increased expression of *Tet2* resulting from OS may be responsible for the abnormal methylation and hydroxymethylation patterns observed in GV and MII oocytes. Previous studies have suggested that the paternal genome is actively demethylated in zygotes, whereas the maternal genome undergoes passive demethylation via DNA replication during cleavage [37, 38]. Active demethylation in the male pronucleus of mouse zygotes was Tet3-mediated [37–39]. This may explain why elevated TET2 levels in OS zygotes did not affect DNA methylation in the male pronucleus. We also found the expression of ERα was significantly increased in the oocytes obtained by OS. These findings indicate that OS-induced DNA demethylation in oocytes may be partially mediated by estrogen signaling.

Parthenogenetic embryos, formed from the activation and diploidization of oocytes, do not contain a paternal genome; the paternally imprinted gene are unmethylated, whereas the maternally imprinted genes are methylated, as in oocytes [48, 49]. Parthenogenetic embryos have been widely studied as effective tool for paternal and maternal imprinting of genes and reproductive problems for a long time [50]. Parthenogenetic embryonic stem cells exhibit imprinting consistent with the exclusively maternal lineage, which shows a typical bimaternal imprinting pattern, and have potential advantages for studying genomic imprinting [40, 44]. These uniparental embryos and cells are valuable for screening parent-of-origin-specific DNA methylation profiles in order to identify novel imprinted CpG sites [51]. However, there are some disadvantages to the use of parthenogenetic samples. Parthenogenetic foetuses die by day 10 of gestation due to a lack of paternal gene expression, which leads to abnormal regulation of differentiation and proliferation, mainly in extra-embryonic lineages [52]. Most parent-of-origin-specific genes, including *Snrpn*, *Mest*, and *Peg3*, were expressed only in Parthenogenetic foetuses; however, some imprinted genes, such as *Asb4* and *Dcn*, showed disrupted parental-specific expression and/or expression levels deviating from the anticipated levels [53, 54]. Parthenogenetic embryos were further analyzed to determine whether OS induced hypomethylation of DNA in oocytes was maintained in pre-implantation embryos. The results of whole-mount immunofluorescence staining indicated a decrease in global 5mC and an increase in global 5hmC from the zygote to blastocyst stage parthenogenetic embryos. It has been reported that the patterns of imprinted gene expression and methylation status in parthenogenetic placentas were similar to their parthenogenetic origin [55]. We further analyzed whether OS-induced hypomethylation of DNA was maintained in the placenta. Consistent with the findings in oocytes and pre-implantation embryos, OS resulted in a decrease in global 5mC and an increase in global 5hmC levels in mid-gestation parthenogenetic placentas. Next, we identified regions that exhibited methylation differences between the parthenogenetic placentas of the control and OS groups using Agilent SureSelectXT mouse Methyl-Seq. We found that the DNA methylation level of the maternal imprinted gene mesoderm-specific transcript homologue (*Mest*) was significantly decreased in the placentas of the OS group. MEST encodes an a/b-hydrolase fold-family enzyme essential for embryonic growth and its DNA methylation is directly inherited from oocytes [2]. Aberrant DNA methylation in the *Mest* imprinting region or its unbalanced expression in the placenta is associated with abnormal placental development in both human and mouse [56, 57]. It has been reported that the DNA methylation of *Mest* is sensitive to ART manipulation as aberrant demethylation of superovulated oocytes was observed in this region in human and mice [2]. Placentas from pregnancies conceived by IVF/ICSI have reduced DNA methylation levels in MEST differentially methylated regions in humans [58]. In the present study, we found that aberrant DNA methylation in the *Mest* imprinting region caused by OS was maintained in the placentas until mid-gestation. These results suggest that loss of methylation in the *Mest* imprinting region may partly account for the deterioration in placental function caused by OS.

We further investigated how OS upregulates the expression of *Tet2* and thus leads to impaired demethylation in GV and MII oocyte. OS results in supraphysiological levels of maternal serum E2, because of the induced simultaneous growth of multiple oocytes. Numerous studies have shown that E2 induces a rapid loss of global DNA methylation by upregulating TET2 in breast cancer cells [35, 36]. Therefore, we evaluated the effect of supraphysiological E2 on 5mC/5hmC and *Tet2* expression in oocytes. Our data revealed that supraphysiological E2 induced a significant decrease in global 5mC and an increase in global 5hmC in GV and MII oocytes. Conversely, the treatment of oocytes with TAM, a selective estrogen receptor degrader (SERD), had the opposite effect on *Tet2* gene expression and 5mC oxidation in oocytes. Moreover, supraphysiological E2 promotes *Tet2* expression. Our findings suggest that supraphysiological E2 can affect DNA methylation by modulating *Tet2* expression, which may participate in the OS induced loss of imprinted methylation in oocytes and early embryos.

Owing to the scarcity of experimental materials, it is difficult to analyze the molecular mechanisms of mouse oocytes. PESCs exhibit imprinting consistent with an exclusively maternal lineage that shows a typical bimaternal imprinting pattern [40, 44]. Therefore, pESCs are a valuable in vitro model for studying the molecular mechanisms underlying DNA methylation and imprinting in oocytes. Under supraphysiological E2 condition, the global levels of 5mC significantly decreased and the global levels of 5hmC significantly increased. Western blotting of TET2 protein levels revealed a steady increase in TET2 protein levels in response to supraphysiological E2 treatment. It has been demonstrated that TET2 is a direct target of E2 in breast cancer cells [35, 36]. As ChIP-qPCR analysis showed, ERα was recruited to the upstream regulatory regions of the *Tet2* gene upon supraphysiological E2 treatment in pESCs. Thus, our data indicate that *Tet2* is a direct transcriptional target gene of ERα, and E2 promotes *Tet2* gene expression at least partially through the direct activation of the ERα function.

Unlike TET1 and TET3, TET2 lacks N-terminal CXXC-type zinc-finger domains, which require co-factors for binding to DNA. Recent studies have demonstrated that TET2 plays a crucial role as a component of the estrogen receptor complex. It is recruited by ERα, which regulates the conversion of 5mC to 5hmC in cis-regulatory regions of the estrogen receptor [35]. This led us to investigate whether TET2 and ERα had the same role in oocytes and pESCs. Similarly, co-immunoprecipitation was conducted and found the physical binding between TET2 and ERα in pESCs under supraphysiological E2. We supposed *Mest* may be the target genes of ERα and ChIP-qPCR analysis showed that ERα directly binds to the *Mest* promoter region. We also found the methylation of *Mest* in pESCs was downregulated by supraphysiological E2 treatment. Knocking down the ERα or TET2 expression or inhibition of ERα in pESCs could rescue the decrease in global 5mC and loss methylation of *Mest* caused by supraphysiological E2. These results indicated the mechanism that TET2 was recruited by ERα to a specific sequence and to convert 5-mC to 5-hmC at specific nucleotides in ERα target genes under supraphysiological E2 in oocyte during OS.

OS results in supraphysiological levels of estradiol, with multiple follicles generated because of the use of high dose of exogenous gonadotropins. Supraphysiological levels of estradiol have adverse effects on endometrial receptivity, as well as obstetric and neonatal outcomes [6, 59–61]. In this study, we revealed that the global decrease in DNA 5mC and increase in 5hmC in mouse oocyte may be caused by supraphysiological E2 during OS. To mitigate this risk, mild stimulation with a lower dose of gonadotropin, can be used to reduce peak estrogen levels without affecting live birth rates. Mild stimulation has been hailed as a safer, patient-friendly approach, with the additional benefit of reducing the cost of gonadotropins [62]. It was also found that adjuvant letrozole in OS could suppress estradiol levels throughout the follicular phase of IVF [63]. Mild stimulation or the use of letrozole in OS may reduce the loss of DNA methylation in oocytes caused by estradiol during ovarian stimulation, increase safety, and reduce epigenetic abnormalities from ART procedures.

This study has some limitations. First, due to technical limitations, we did not verify whether estrogen affects the methylation of *Mest* through the ERα-Tet2 axis in the oocyte. Secondly, most mouse parthenogenetic embryos do not survive beyond day 9.5 of pregnancy, and have very limited development of extraembryonic tissues, including the placenta [53]. Therefore, the placental sample obtained at 10.5 d of pregnancy was very small, and we did not have more samples for histological analysis. Therefore, no detect were detected in the parthenogenetic placenta.

## Conclusions

In conclusion, the results obtained in this study demonstrate for the first time that estrogen signaling play a role in the dysregulation of genomic imprinting in oocytes during OS. The supraphysiological level of E2 caused by OS results in a significant decrease in global 5mC by promoting *Tet2* expression in oocytes. First, E2 promotes *Tet2* gene expression at least partially through direct activation of the ERα. Second, TET2 is recruited by ERα to bind with its target genes and induce DNA demethylation of the target genes. Overall, our findings provide important new insights into the dysregulation of genomic imprinting during oogenesis caused by OS and suggest a new strategy for the prevention of aberrant DNA methylation in oocytes and ultimately to conceive healthier children with the aid of ART.

### Supplementary Information

** Additional file 1: Supplementary Table 1.** The composition of the N2B27 medium.

** Additional file 2: Supplementary Table 2.** Sequences of the primers used in bisulfite sequencing PCR (BSP).

** Additional file 3: Supplementary Table 3.** Sequences of the primers used in quantitative real-time polymerase chain reaction (qRT-PCR).

** Additional file 4: Supplementary Table 4.** Sequences of the primers used in siRNAs.

** Additional file 5: Supplementary Table 5.** Sequences of the primers used in Chromatin immunoprecipitation-PCR (CHIP -PCR).

** Additional file 6: Supplementary Fig. 1.** Effect of ovarian stimulation (OS) on β-estradiol (E2) level, 5-methylcytosine (5mC) and 5-hydroxymethylcytosine (5hmC) of pregnant mice. A: The serum E2 level of mice within each group (*n* = 6). B, C: The placental 5mC, 5hmC percentage and the 5hmC/5mC ratio of E10.5 placenta were detected by Enzyme-Linked Immunosorbent Assay (ELISA) kit (*n* = 9). Data are expressed as the means ± standard deviation (SD), *P < 0.05, **P < 0.01, ***P < 0.001. (Unpaired two-tailed t test was used to compare differences among two groups and one-way ANOVA multiple comparisons test was performed among the three groups).

** Additional file 7: Supplementary Fig. 2.** Experimental flow diagram of Agilent SureSelectXT mouse Methyl-Seq.

** Additional file 8: Supplementary Fig. 3.** A, B: Analysis of methylation levels at cytosine preceding a guanine base (CpG) sites within the differentially methylated region (DMR) in the Pleiomorphic Adenoma Gene-Like 1(*Plagl1*) promoter were used by bisulfite sequencing PCR (BSP, *n* = 9). C: Chromatin immunoprecipitation (ChIP) assay using ERα as bait protein demonstrated the interaction between ERα and *Plagl1* (*n* = 3). Data are expressed as the means ± standard deviation (SD), ns: Not Statistically Significant (unpaired two-tailed t test).

** Additional file 9: Supplementary Fig. 4.** Analysis of RNA levels for *Mest* was performed within each group (*n* = 8). Data are expressed as the means ± standard deviation (SD), **P < 0.01 (unpaired two-tailed t test).

** Additional file 10: Supplementary Fig. 5. A, B:** Quantification of estrogen receptor alpha (*Esr1*) and ten-eleven translocation 2(*Tet2*) expression levels within each group by quantitative real-time polymerase chain reaction (qRT-PCR, *n* = 3). Data are expressed as the means ± standard deviation (SD), ns: Not Statistically Significant, *P < 0.05, **P < 0.01 (unpaired two-tailed t test).

** Additional file 11.**

## Data Availability

The main data supporting the finds of this study are available within the article.

## Abbreviations

OS: Ovarian stimulation
ART: Assisted reproductive technologies
LBW: Low birth weight
pESCs: Parthenogenetic embryonic stem cells
E2: β- estradiol
hCG: Human chorionic gonadotropin
MEST: Mesoderm-specific transcript homologue
BSP: Bisulfite sequencing PCR
ERα, ESR1: Estrogen receptor alpha
TET2: Ten-eleven translocation 2
DNMTs: DNA methyltransferases
5fC: 5-Formylcytosine
5caC: 5-Carboxylcytosine
CpG: Cytosine preceding a guanine base
TAM: TAMXIFEN
ALP: Alkaline phosphatase
qRT-PCR: Quantitative real-time polymerase chain reaction
ChIP: Chromatin immunoprecipitation
Co-IP: Co-inmunoprecipitation
GV: Germinal vesicle
MII: Metaphase II
Gd: Gestational day
PVDF: Polyvinylidene fluoride
TBST: Tris Buffered Saline with Tween 20

## References

[1] Sciorio R, Tramontano L, Rapalini E, Bellaminutti S, Bulletti FM, D’Amato A (2023). Risk of genetic and epigenetic alteration in children conceived following ART: is it time to return to nature whenever possible?. Clin Genet.

[2] Barberet J, Ducreux B, Guilleman M, Simon E, Bruno C, Fauque P (2022). DNA methylation profiles after ART during human lifespan: a systematic review and meta-analysis. Hum Reprod Update.

[3] Schieve LA, Meikle SF, Ferre C, Peterson HB, Jeng G, Wilcox LS (2002). Low and very low birth weight in infants conceived with use of assisted reproductive technology. N Engl J Med.

[4] Kalra SK, Ratcliffe SJ, Coutifaris C, Molinaro T, Barnhart KT (2011). Ovarian stimulation and low birth weight in newborns conceived through in vitro fertilization. Obstet Gynecol.

[5] Hu X-L, Feng C, Lin X-H, Zhong Z-X, Zhu Y-M, Lv P-P (2014). High maternal serum estradiol environment in the first trimester is associated with the increased risk of small-for-gestational-age birth. J Clin Endocrinol Metab.

[6] Pereira N, Elias RT, Christos PJ, Petrini AC, Hancock K, Lekovich JP (2017). Supraphysiologic estradiol is an independent predictor of low birth weight in full-term singletons born after fresh embryo transfer. Hum Reprod.

[7] Poulain M, de Ziegler D, Ayoubi JM (2019). Epigenetic alterations of the first trimester placenta: insight into preoccupying concerns in assisted reproductive technology. Fertil Steril.

[8] de Waal E, Vrooman LA, Fischer E, Ord T, Mainigi MA, Coutifaris C (2015). The cumulative effect of assisted reproduction procedures on placental development and epigenetic perturbations in a mouse model. Hum Mol Genet.

[9] Ganer Herman H, Volodarsky-Perel A, Ton Nu TN, Machado-Gedeon A, Cui Y, Shaul J (2022). The effect of higher estradiol levels during stimulation on pregnancy complications and placental histology. Placenta..

[10] Ma R, Jin N, Lei H, Dong J, Xiong Y, Qian C (2023). Ovarian stimulation in mice resulted in abnormal placentation through its effects on proliferation and cytokine production of uterine NK cells. Int J Mol Sci.

[11] Zhang W, Ma Y, Xiong Y, Xiao X, Chen S, Wang X (2019). Supraphysiological serum oestradiol negatively affects birthweight in cryopreserved embryo transfers: a retrospective cohort study. Reprod Biomed Online.

[12] Cai J, Liu L, Xu Y, Liu Z, Jiang X, Li P (2019). Supraphysiological estradiol level in ovarian stimulation cycles affects the birthweight of neonates conceived through subsequent frozen-thawed cycles: a retrospective study. BJOG..

[13] Håberg SE, Page CM, Lee Y, Nustad HE, Magnus MC, Haftorn KL (2022). DNA methylation in newborns conceived by assisted reproductive technology. Nat Commun.

[14] Mani S, Ghosh J, Coutifaris C, Sapienza C, Mainigi M (2020). Epigenetic changes and assisted reproductive technologies. Epigenetics..

[15] Li B, Chen S, Tang N, Xiao X, Huang J, Jiang F (2016). Assisted reproduction causes reduced fetal growth associated with downregulation of paternally expressed imprinted genes that enhance fetal growth in mice. Biol Reprod.

[16] Chen S, Sun F, Huang X, Wang X, Tang N, Zhu B (2015). Assisted reproduction causes placental maldevelopment and dysfunction linked to reduced fetal weight in mice. Sci Rep.

[17] Tomizawa S-I, Nowacka-Woszuk J, Kelsey G (2012). DNA methylation establishment during oocyte growth: mechanisms and significance. Int J Dev Biol.

[18] Sato A, Otsu E, Negishi H, Utsunomiya T, Arima T (2007). Aberrant DNA methylation of imprinted loci in superovulated oocytes. Hum Reprod.

[19] Market-Velker BA, Zhang L, Magri LS, Bonvissuto AC, Mann MRW (2010). Dual effects of superovulation: loss of maternal and paternal imprinted methylation in a dose-dependent manner. Hum Mol Genet.

[20] Yu B, Smith TH, Battle SL, Ferrell S, Hawkins RD (2019). Superovulation alters global DNA methylation in early mouse embryo development. Epigenetics..

[21] Velker BAM, Denomme MM, Krafty RT, Mann MRW (2017). Maintenance of Mest imprinted methylation in blastocyst-stage mouse embryos is less stable than other imprinted loci following superovulation or embryo culture. Environ Epigenet.

[22] Saenz-de-Juano MD, Billooye K, Smitz J, Anckaert E (2016). The loss of imprinted DNA methylation in mouse blastocysts is inflicted to a similar extent by in vitro follicle culture and ovulation induction. Mol Hum Reprod.

[23] Huffman SR, Pak Y, Rivera RM (2015). Superovulation induces alterations in the epigenome of zygotes, and results in differences in gene expression at the blastocyst stage in mice. Mol Reprod Dev.

[24] Branco MR, King M, Perez-Garcia V, Bogutz AB, Caley M, Fineberg E (2016). Maternal DNA methylation regulates early trophoblast development. Dev Cell.

[25] Sendžikaitė G, Kelsey G (2019). The role and mechanisms of DNA methylation in the oocyte. Essays Biochem.

[26] Joshi K, Liu S, Breslin SJP, Zhang J (2022). Mechanisms that regulate the activities of TET proteins. Cell Mol Life Sci.

[27] Wu X, Zhang Y (2017). TET-mediated active DNA demethylation: mechanism, function and beyond. Nat Rev Genet.

[28] Denomme MM, Zhang L, Mann MRW (2011). Embryonic imprinting perturbations do not originate from superovulation-induced defects in DNA methylation acquisition. Fertil Steril.

[29] Marshall KL, Rivera RM (2018). The effects of superovulation and reproductive aging on the epigenome of the oocyte and embryo. Mol Reprod Dev.

[30] Liu Y, Rosikiewicz W, Pan Z, Jillette N, Wang P, Taghbalout A (2021). DNA methylation-calling tools for Oxford Nanopore sequencing: a survey and human epigenome-wide evaluation. Genome Biol.

[31] Zhang X, Zhang Y, Wang C, Wang X (2023). TET (ten-eleven translocation) family proteins: structure, biological functions and applications. Signal Transduct Target Ther.

[32] Wang H, Zhou C, Chen W, Li T, Huang J, Zhuang G (2011). Supraphysiological estrogen levels adversely impact proliferation and histone modification in human embryonic stem cells: possible implications for controlled ovarian hyperstimulation assisted pregnancy. Eur J Obstet Gynecol Reprod Biol.

[33] Kovalchuk O, Tryndyak VP, Montgomery B, Boyko A, Kutanzi K, Zemp F (2007). Estrogen-induced rat breast carcinogenesis is characterized by alterations in DNA methylation, histone modifications and aberrant microRNA expression. Cell Cycle.

[34] Zhu X, Xiong L, Lyu R, Shen Y, Liu L, Li S (2022). Regulation of TET2 gene expression and 5mC oxidation in breast cancer cells by estrogen signaling. Biochem Biophys Res Commun.

[35] Broome R, Chernukhin I, Jamieson S, Kishore K, Papachristou EK, Mao S-Q (2021). TET2 is a component of the estrogen receptor complex and controls 5mC to 5hmC conversion at estrogen receptor cis-regulatory regions. Cell Rep.

[36] Wang L, Ozark PA, Smith ER, Zhao Z, Marshall SA, Rendleman EJ (2018). TET2 coactivates gene expression through demethylation of enhancers. Sci Adv.

[37] Ooi SKT, Bestor TH (2008). The colorful history of active DNA demethylation. Cell..

[38] Gu T-P, Guo F, Yang H, Wu H-P, Xu G-F, Liu W (2011). The role of Tet3 DNA dioxygenase in epigenetic reprogramming by oocytes. Nature..

[39] Chen Y, Wang L, Guo F, Dai X, Zhang X (2020). Epigenetic reprogramming during the maternal-to-zygotic transition. MedComm.

[40] Tian C, Liu L, Zeng M, Sheng X, Heng D, Wang L (2021). Generation of developmentally competent oocytes and fertile mice from parthenogenetic embryonic stem cells. Protein Cell.

[41] Akbar R, Ullah K, Rahman TU, Cheng Y, Pang H-Y, Jin L-Y (2020). miR-183-5p regulates uterine receptivity and enhances embryo implantation. J Mol Endocrinol.

[42] Ullah K, Rahman TU, Pan H-T, Guo M-X, Dong X-Y, Liu J (2017). Serum estradiol levels in controlled ovarian stimulation directly affect the endometrium. J Mol Endocrinol.

[43] De Jaime-Soguero A, Aulicino F, Ertaylan G, Griego A, Cerrato A, Tallam A (2017). Wnt/Tcf1 pathway restricts embryonic stem cell cycle through activation of the Ink4/Arf locus. PLoS Genet.

[44] Seo BJ, Jang HS, Song H, Park C, Hong K, Lee JW (2019). Generation of mouse parthenogenetic epiblast stem cells and their imprinting patterns. Int J Mol Sci.

[45] Dumasia K, Kumar A, Deshpande S, Balasinor NH (2017). Estrogen signaling, through estrogen receptor β, regulates DNA methylation and its machinery in male germ line in adult rats. Epigenetics..

[46] Canovas S, Ivanova E, Romar R, García-Martínez S, Soriano-Úbeda C, García-Vázquez FA (2017). DNA methylation and gene expression changes derived from assisted reproductive technologies can be decreased by reproductive fluids. Elife..

[47] Chen W, Peng Y, Ma X, Kong S, Tan S, Wei Y (2020). Integrated multi-omics reveal epigenomic disturbance of assisted reproductive technologies in human offspring. EBioMedicine..

[48] Wei M, Zhang J, Liu J, Zhao C, Cao S, Yan X (2021). Derivation of mouse parthenogenetic advanced stem cells. Int J Mol Sci.

[49] Wei Y, Yang C-R, Zhao Z-A (2022). Viable offspring derived from single unfertilized mammalian oocytes. Proc Natl Acad Sci USA.

[50] Chu C, Zhang W, Kang Y, Si C, Ji W, Niu Y (2021). Analysis of developmental imprinting dynamics in primates using SNP-free methods to identify imprinting defects in cloned placenta. Dev Cell.

[51] Choi NY, Bang JS, Lee HJ, Park YS, Lee M, Jeong D (2018). Novel imprinted single CpG sites found by global DNA methylation analysis in human parthenogenetic induced pluripotent stem cells. Epigenetics..

[52] Kono T, Obata Y, Yoshimzu T, Nakahara T, Carroll J (1996). Epigenetic modifications during oocyte growth correlates with extended parthenogenetic development in the mouse. Nat Genet.

[53] Ogawa H, Shindo N, Kumagai T, Usami Y, Shikanai M, Jonwn K (2009). Developmental ability of trophoblast stem cells in uniparental mouse embryos. Placenta..

[54] Ogawa H, Wu Q, Komiyama J, Obata Y, Kono T (2006). Disruption of parental-specific expression of imprinted genes in uniparental fetuses. FEBS Lett.

[55] Hikichi T, Ohta H, Wakayama S, Wakayama T (2010). Functional full-term placentas formed from parthenogenetic embryos using serial nuclear transfer. Development..

[56] Peng W, Chen Y, Luo X, Shan N, Lan X, Olson D (2016). DNA methylation-associated repression of MEST/PEG1 expression contributes to the invasion of extravillous trophoblast cells. Placenta..

[57] Himes KP, Young A, Koppes E, Stolz D, Barak Y, Sadovsky Y (2015). Loss of inherited genomic imprints in mice leads to severe disruption in placental lipid metabolism. Placenta..

[58] Nelissen ECM, Dumoulin JCM, Daunay A, Evers JLH, Tost J, van Montfoort APA (2013). Placentas from pregnancies conceived by IVF/ICSI have a reduced DNA methylation level at the H19 and MEST differentially methylated regions. Hum Reprod.

[59] Pereira N, Reichman DE, Goldschlag DE, Lekovich JP, Rosenwaks Z (2015). Impact of elevated peak serum estradiol levels during controlled ovarian hyperstimulation on the birth weight of term singletons from fresh IVF-ET cycles. J Assist Reprod Genet.

[60] Davies MJ, Moore VM, Willson KJ, Van Essen P, Priest K, Scott H (2012). Reproductive technologies and the risk of birth defects. N Engl J Med.

[61] Simon C, Domínguez F, Valbuena D, Pellicer A (2003). The role of estrogen in uterine receptivity and blastocyst implantation. Trends Endocrinol Metab.

[62] Datta AK, Maheshwari A, Felix N, Campbell S, Nargund G (2021). Mild versus conventional ovarian stimulation for IVF in poor, normal and hyper-responders: a systematic review and meta-analysis. Hum Reprod Update.

[63] Dreyer Holt M, Skouby SO, Bülow NS, Englund ALM, Birch Petersen K, Macklon NS (2022). The impact of suppressing estradiol during ovarian stimulation on the unsupported luteal phase: a randomized controlled trial. J Clin Endocrinol Metab.

