# Supplementary material for: High estrogen during ovarian stimulation induced loss of maternal imprinted methylation that is essential for placental development via overexpression of TET2 in mouse oocytes
Source: Cell Commun Signal. 2024 Feb 19;22:135. doi: 10.1186/s12964-024-01516-x (PMC10875811; doi:10.1186/s12964-024-01516-x)
Supplement: Supplementary file 1 — Additional file 1: Supplementary Table 1. The composition of the N2B27 medium. [file 12964_2024_1516_MOESM1_ESM.docx]

Supplementary Table 1 The composition of the N2B27 medium.

| Component | Volume/mL | Company |
| --- | --- | --- |
| DMEM/F12 medium | 23.75 | Gibco |
| Neurobasal medium | 23.75 | Gibco |
| N2 supplement | 0.5 | Thermo |
| B27 supplement | 1 | Thermo |
| Bovine Serum Albumin（7.5%） | 0.033 | Thermo |
| MEM NEAA | 1 | Thermo |
| L-glutamax | 1 | Thermo |
| 2-Mercaptoethanol | 0.05 | Gibco |
| Penicillin-Streptomycin | 0.5 | Absin |
