# Supplementary material for: High estrogen during ovarian stimulation induced loss of maternal imprinted methylation that is essential for placental development via overexpression of TET2 in mouse oocytes
Source: Cell Commun Signal. 2024 Feb 19;22:135. doi: 10.1186/s12964-024-01516-x (PMC10875811; doi:10.1186/s12964-024-01516-x)
Supplement: Supplementary file 2 — Additional file 2: Supplementary Table 2. Sequences of the primers used in bisulfite sequencing PCR (BSP). [file 12964_2024_1516_MOESM2_ESM.docx]

Supplementary Table 2 Sequences of the primers used in bisulfite sequencing PCR (BSP).

| Gene | Left (5’-3’) | Right (5’-3’) |
| --- | --- | --- |
| *Mest* | *TTATGGTATAGAAGGATGTGAAGGTAA* | *AACCCCTTAAAACACCTAAAAAAAC* |
| *Plagl1* | *AAAGGTTTTAAAAATGTTGGGAATT* | *AACTCCAACCCAAACCTATAAATTAA* |
