# Supplementary material for: High estrogen during ovarian stimulation induced loss of maternal imprinted methylation that is essential for placental development via overexpression of TET2 in mouse oocytes
Source: Cell Commun Signal. 2024 Feb 19;22:135. doi: 10.1186/s12964-024-01516-x (PMC10875811; doi:10.1186/s12964-024-01516-x)
Supplement: Supplementary file 3 — Additional file 3: Supplementary Table 3. Sequences of the primers used in quantitative real-time polymerase chain reaction (qRT-PCR). [file 12964_2024_1516_MOESM3_ESM.docx]

Supplementary Table 3 Sequences of the primers used in quantitative real-time polymerase chain reaction (qRT-PCR).

| Gene | Forward (5’-3’) | Reverse (5’-3’) |
| --- | --- | --- |
| *Actin* | *GAAATCGTGCGTGACATCAAAG* | *TGTAGTTTCATGGATGCCACAG* |
| *Dnmt1* | *TTCCACCAAGCAGGCATCTC* | *TTTGGCCCAGAGAGTTGGTC* |
| *Dnmt3a* | *GGCCTTCTCGACTCCAGATG* | *TTCCTCTTCTCAGCTGGCAC* |
| *Dnmt3b* | *AATACCCAACTCCTTGAGCAC* | *TCTTCACTACTGATCCTGACCT* |
| *Tet1* | *GAGAGATTTCTCGGGTCAGCAT* | *TTCCTCCTCTCCACCATTGG* |
| *Tet2* | *GGCAAATGTGAAGGATGCAA* | *CCAGCTCCTAGATGGGTATAATAAGG* |
| *Tet3* | *GGGCAGGCAGCGTAGC* | *ATGAGGTGAGCCAATGGGTG* |
| *ER alpha* | *AATTCTGACAATCGACGCCAG* | *GTGCTTCAACATTCTCCCTCCTC* |
| *Mest* | *GTGGTGGGTCCAAGTAGGG* | *AAGCACAACTATCTCAGGGCT* |
| *Plagl1* | *ATGGCTCCATTCCGCTGTC* | *CTCAGCCTTCGAGCACTTGAA* |
