# Supplementary material for: High estrogen during ovarian stimulation induced loss of maternal imprinted methylation that is essential for placental development via overexpression of TET2 in mouse oocytes
Source: Cell Commun Signal. 2024 Feb 19;22:135. doi: 10.1186/s12964-024-01516-x (PMC10875811; doi:10.1186/s12964-024-01516-x)
Supplement: Supplementary file 4 — Additional file 4: Supplementary Table 4. Sequences of the primers used in siRNAs. [file 12964_2024_1516_MOESM4_ESM.docx]

Supplementary Table 4 Sequences of the primers used in siRNAs.

| Gene | Sense (5’-3’) | Antisense (5’-3’) |
| --- | --- | --- |
| *siTet2-1* | *GGCUGUCAAACUCCAGAAUTT* | *AUUCUGGAGUUUGACAGCCTT* |
| *siTet2-2* | *GCCUCGGGUUCAUAUUUGATT* | *UCAAAUAUGAACCCGAGGCTT* |
| *siTet2-3* | *CAGGGAUCUACAUAGAUAUTT* | *AUAUCUAUGUAGAUCCCUGTT* |
| *siTet2-4* | *GGCCUGUGAUGCUGAUAAUTT* | *AUUAUCAGCAUCACAGGCCTT* |
| *siEsr1-1* | *GGGAGCUGGUUCAUAUGAUTT* | *AUCAUAUGAACCAGCUCCCTT* |
| *siEsr1-2* | *GGCUGGAGAUUCUGAUGAUTT* | *AUCAUCAGAAUCUCCAGCCTT* |
| *siEsr1-3* | *GUCCAGCAGUAACGAGAAATT* | *UUUCUCGUUACUGCUGGACTT* |
| *siEsr1-4* | *CAAGCCCUCUUGUGAUUAATT* | *UUAAUCACAAGAGGGCUUGTT* |
