# Supplementary material for: High estrogen during ovarian stimulation induced loss of maternal imprinted methylation that is essential for placental development via overexpression of TET2 in mouse oocytes
Source: Cell Commun Signal. 2024 Feb 19;22:135. doi: 10.1186/s12964-024-01516-x (PMC10875811; doi:10.1186/s12964-024-01516-x)
Supplement: Supplementary file 5 — Additional file 5: Supplementary Table 5. Sequences of the primers used in Chromatin immunoprecipitation-PCR (CHIP -PCR). [file 12964_2024_1516_MOESM5_ESM.docx]

Supplementary Table 5 Sequences of the primers used in Chromatin immunoprecipitation-PCR (CHIP -PCR).

| Gene | Forward (5’-3’) | Reverse (5’-3’) |
| --- | --- | --- |
| *Mest* | *AGCATTTTGGATGCCAGTCC* | *AGCAAGTTTCCGTTTCCTTTG* |
| *Plagl1* | *TGCCAAATTCCTTGGCCTCT* | *GCCACATCGCAAACCAGAAA* |
