# Supplementary figures and images for: High estrogen during ovarian stimulation induced loss of maternal imprinted methylation that is essential for placental development via overexpression of TET2 in mouse oocytes
Source: Cell Commun Signal. 2024 Feb 19;22:135. doi: 10.1186/s12964-024-01516-x (PMC10875811; doi:10.1186/s12964-024-01516-x)

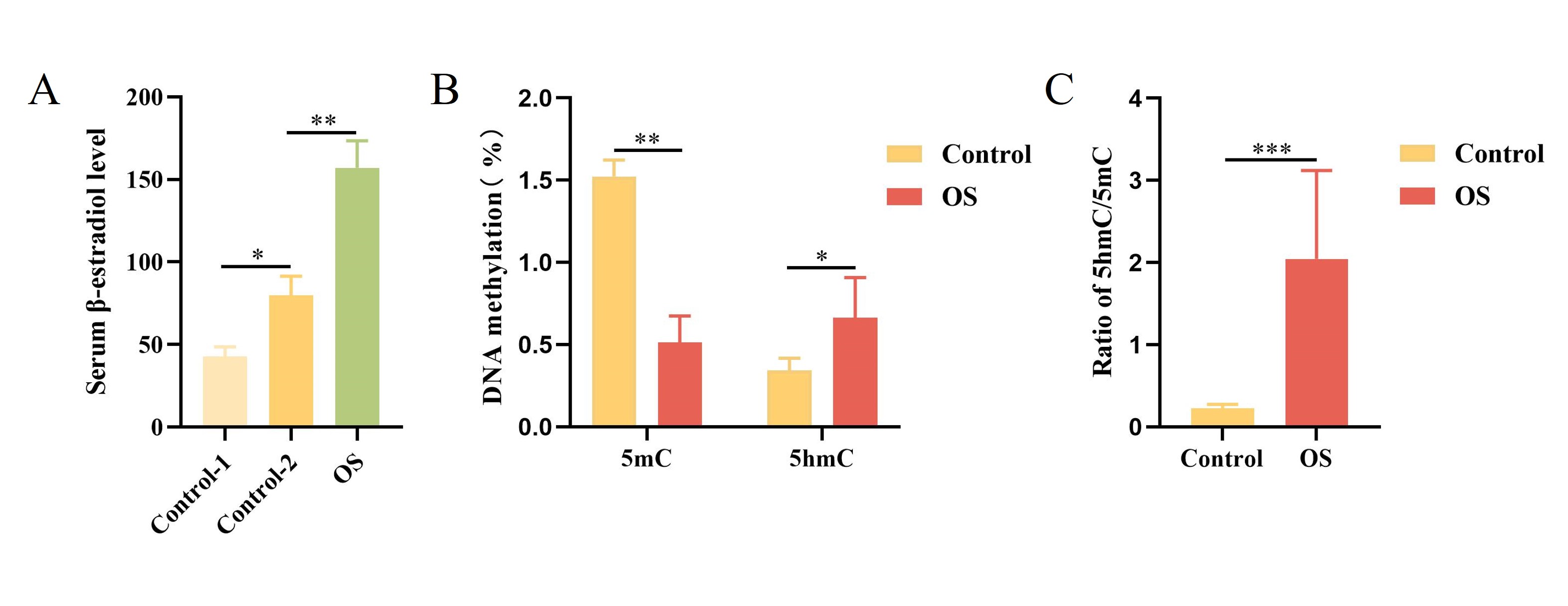

Supplement: Supplementary file 6 — Additional file 6: Supplementary Fig. 1. Effect of ovarian stimulation (OS) on β-estradiol (E2) level, 5-methylcytosine (5mC) and 5-hydroxymethylcytosine (5hmC) of pregnant mice. A: The serum E2 level of mice within each group (n = 6). B, C: The placental 5mC, 5hmC percentage and the 5hmC/5mC ratio of E10.5 placenta were detected by Enzyme-Linked Immunosorbent Assay (ELISA) kit (n = 9). Data are expressed as the means ± standard deviation (SD), *P < 0.05, **P < 0.01, ***P < 0.001. (Unpaired two-tailed t test was used to compare differences among two groups and one-way ANOVA multiple comparisons test was performed among the three groups). [file 12964_2024_1516_MOESM6_ESM.jpg]

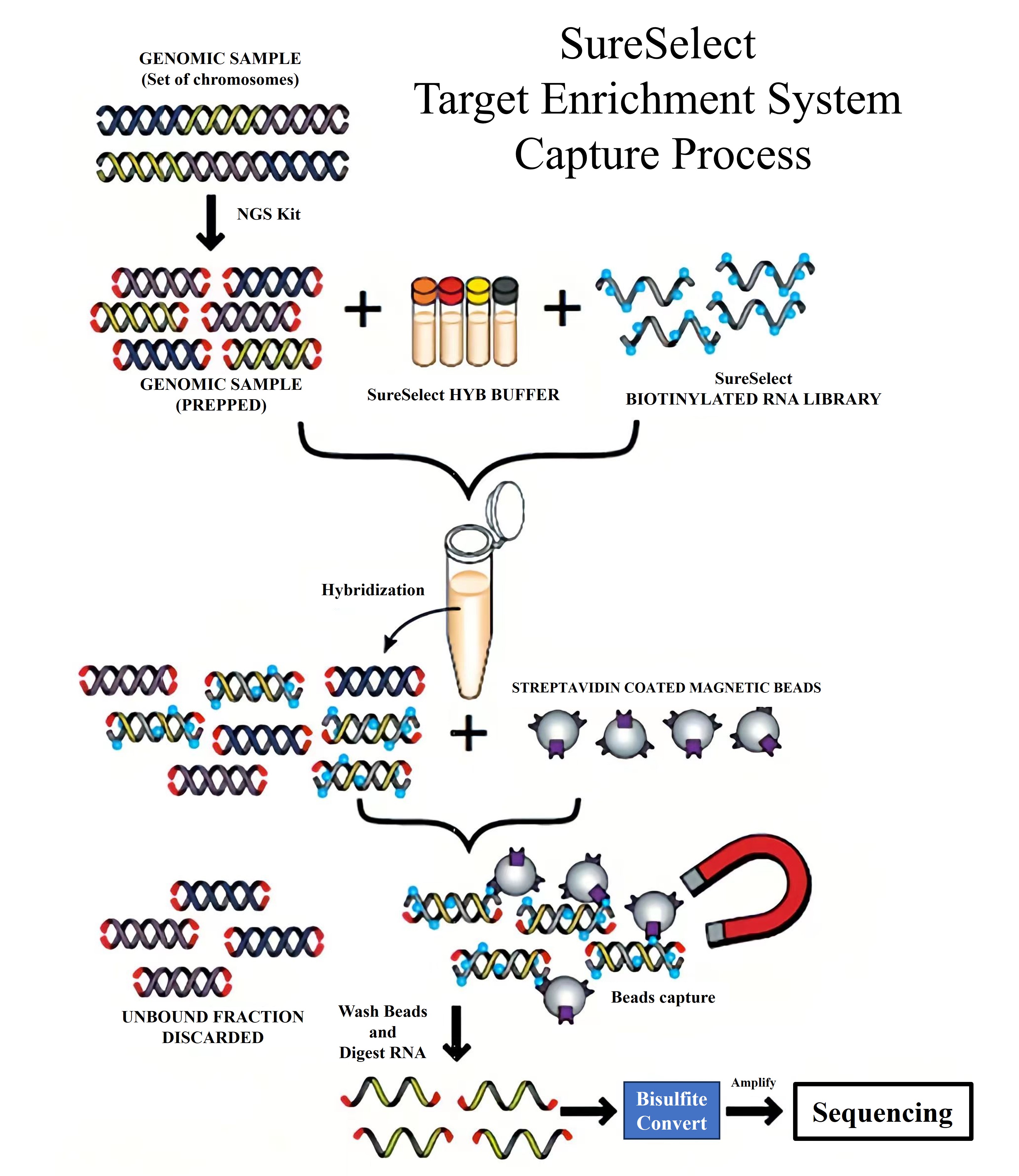

Supplement: Supplementary file 7 — Additional file 7: Supplementary Fig. 2. Experimental flow diagram of Agilent SureSelectXT mouse Methyl-Seq. [file 12964_2024_1516_MOESM7_ESM.jpg]

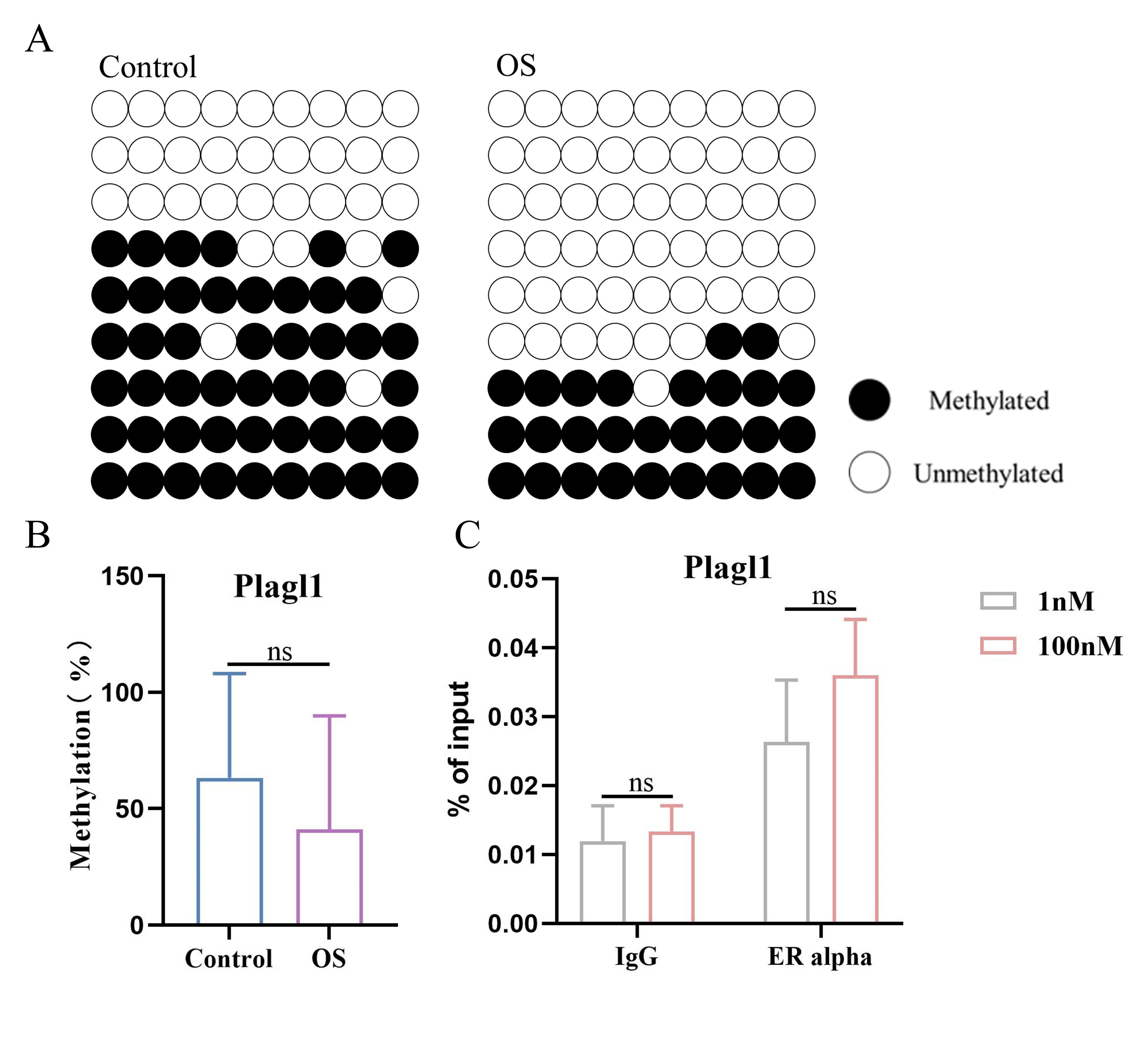

Supplement: Supplementary file 8 — Additional file 8: Supplementary Fig. 3. A, B: Analysis of methylation levels at cytosine preceding a guanine base (CpG) sites within the differentially methylated region (DMR) in the Pleiomorphic Adenoma Gene-Like 1(Plagl1) promoter were used by bisulfite sequencing PCR (BSP, n = 9). C: Chromatin immunoprecipitation (ChIP) assay using ERα as bait protein demonstrated the interaction between ERα and Plagl1 (n = 3). Data are expressed as the means ± standard deviation (SD), ns: Not Statistically Significant (unpaired two-tailed t test). [file 12964_2024_1516_MOESM8_ESM.jpg]

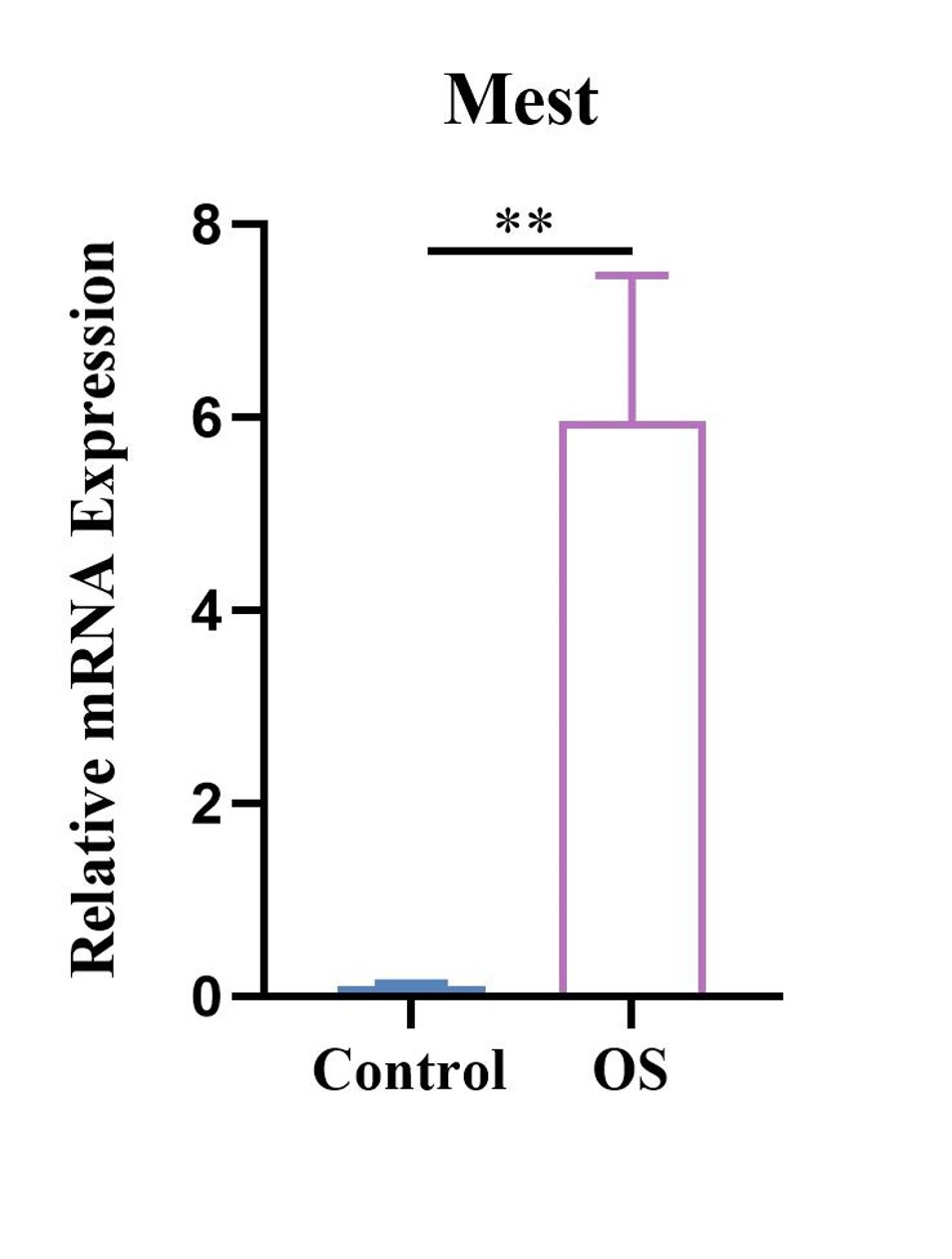

Supplement: Supplementary file 9 — Additional file 9: Supplementary Fig. 4. Analysis of RNA levels for Mest was performed within each group (n = 8). Data are expressed as the means ± standard deviation (SD), **P < 0.01 (unpaired two-tailed t test). [file 12964_2024_1516_MOESM9_ESM.jpg]

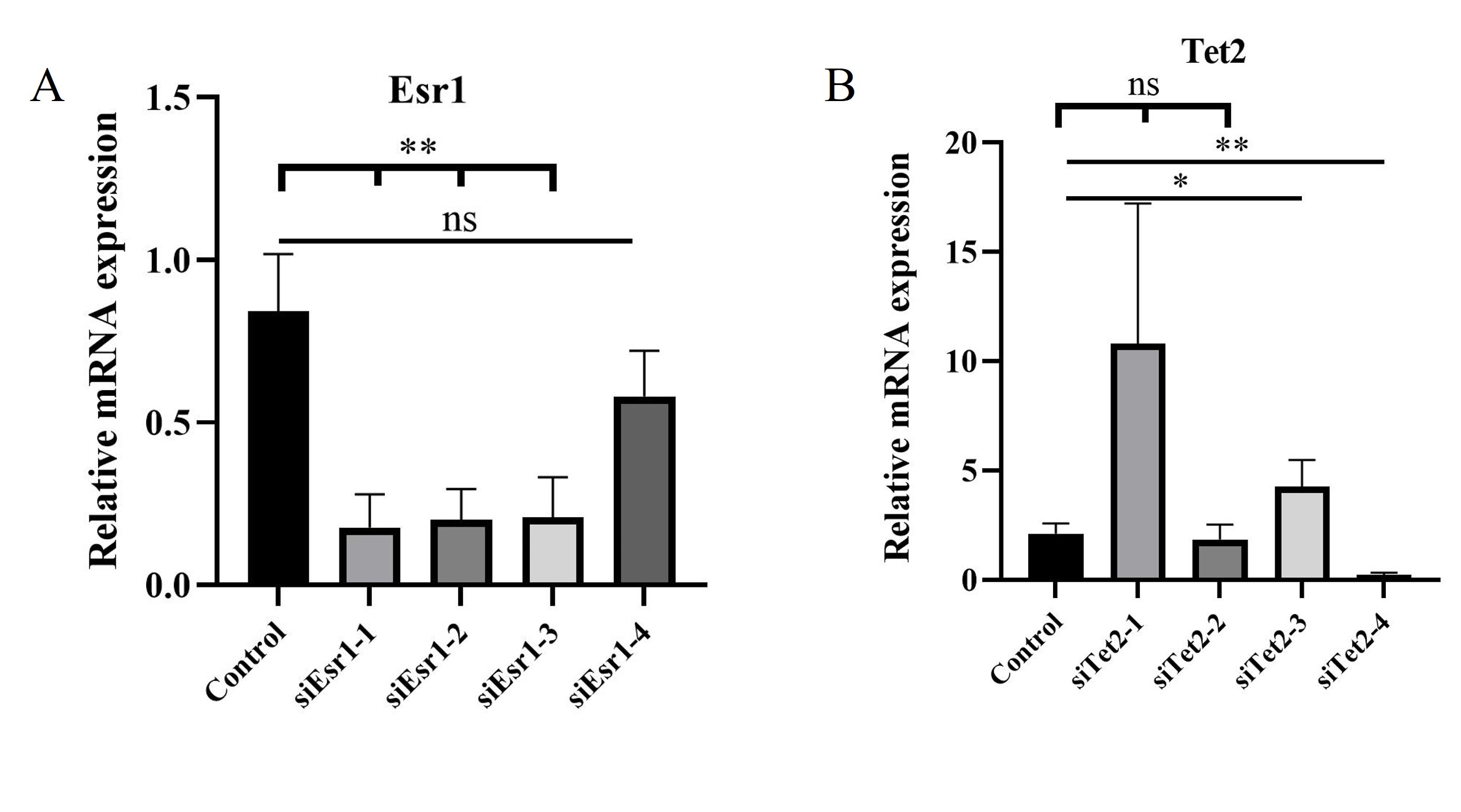

Supplement: Supplementary file 10 — Additional file 10: Supplementary Fig. 5. A, B: Quantification of estrogen receptor alpha (Esr1) and ten-eleven translocation 2(Tet2) expression levels within each group by quantitative real-time polymerase chain reaction (qRT-PCR, n = 3). Data are expressed as the means ± standard deviation (SD), ns: Not Statistically Significant, *P < 0.05, **P < 0.01 (unpaired two-tailed t test). [file 12964_2024_1516_MOESM10_ESM.jpg]
