# Supplementary material for: High estrogen during ovarian stimulation induced loss of maternal imprinted methylation that is essential for placental development via overexpression of TET2 in mouse oocytes
Source: Cell Commun Signal. 2024 Feb 19;22:135. doi: 10.1186/s12964-024-01516-x (PMC10875811; doi:10.1186/s12964-024-01516-x)

MEST-cell


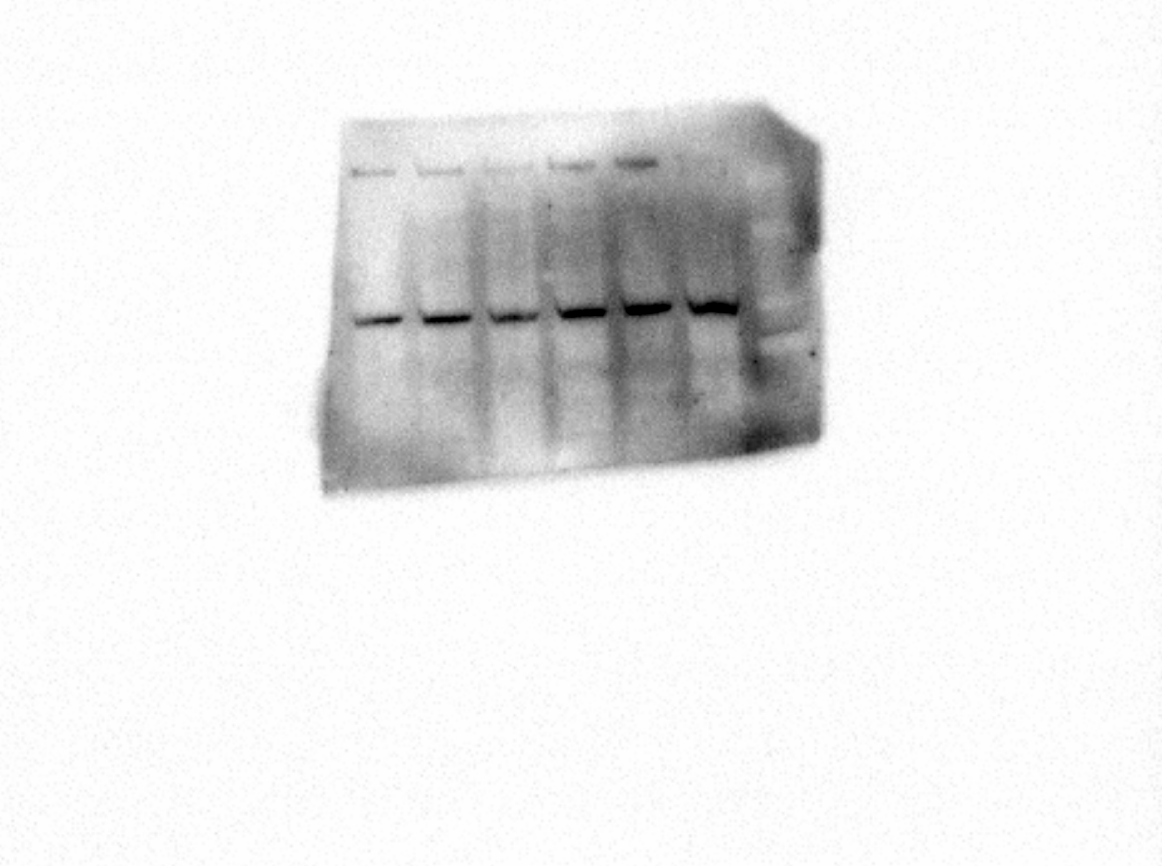


TET2-Actin-cell


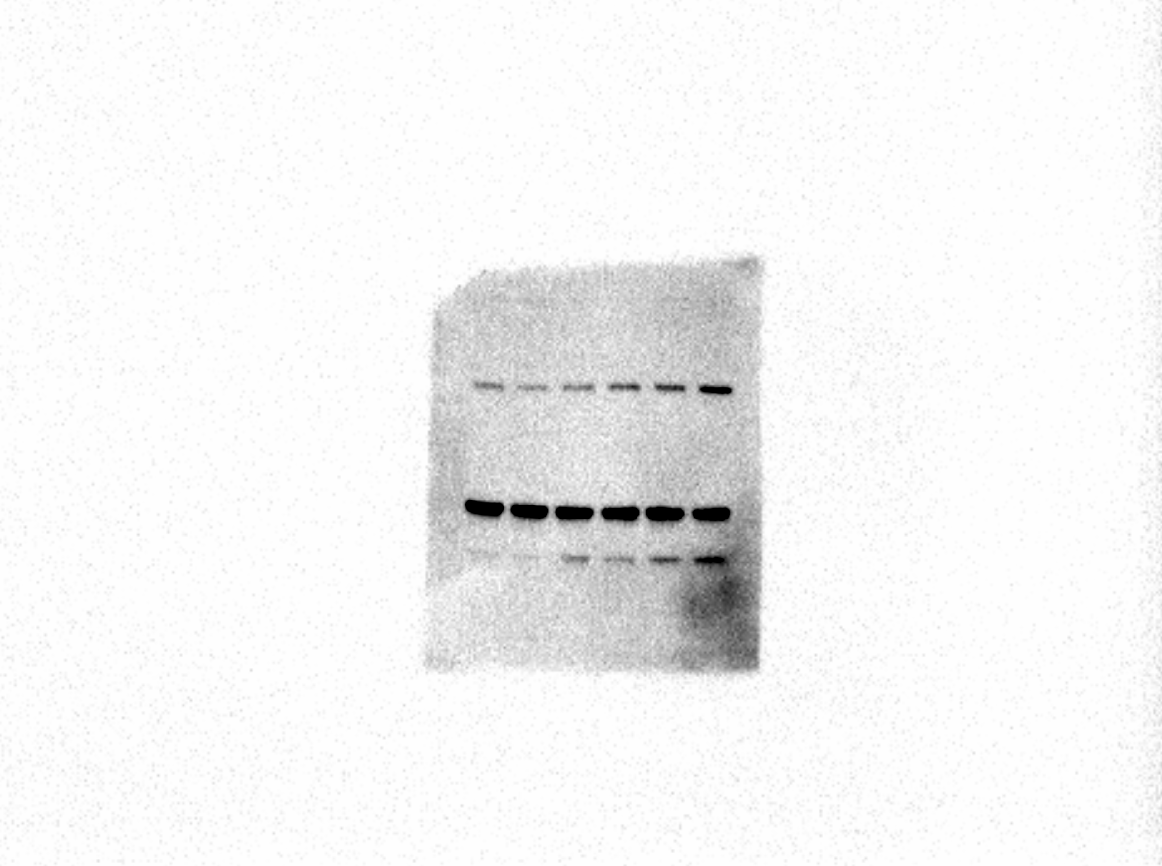


ER alpha-cell


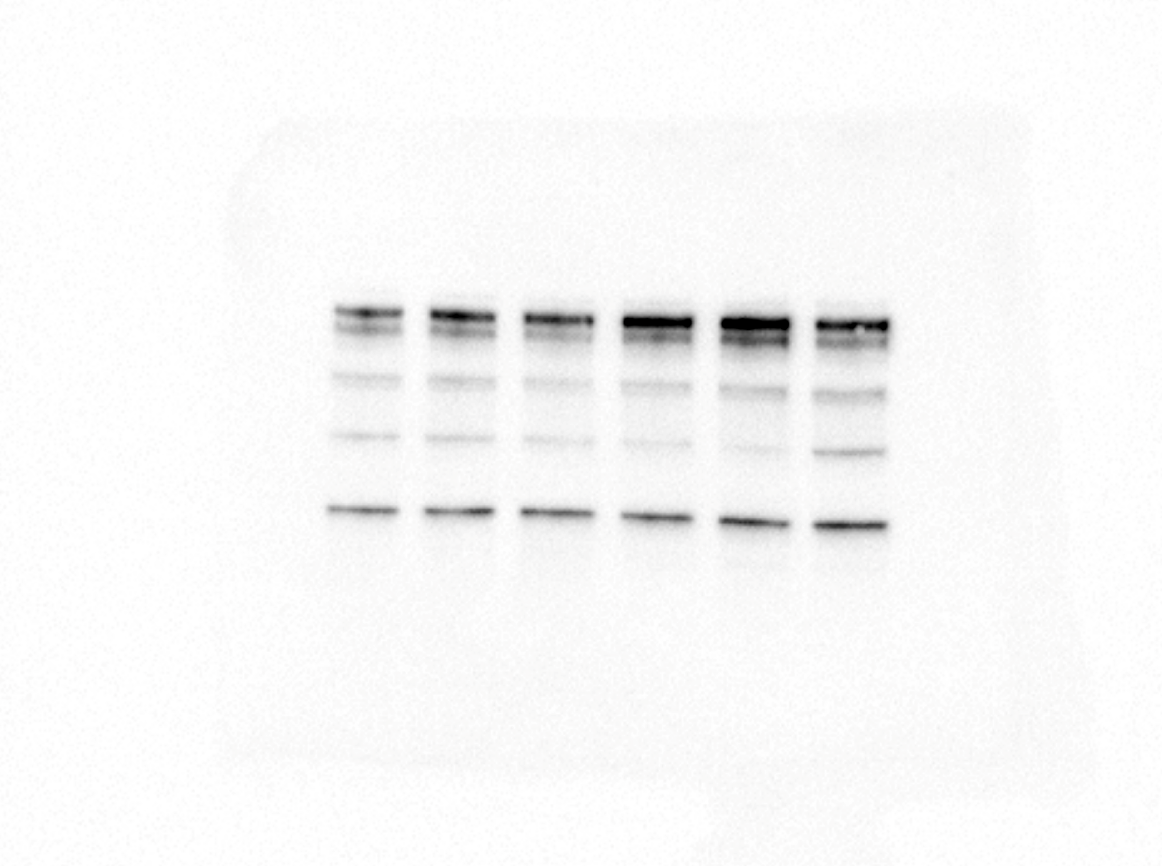


TET2-Actin-placenta


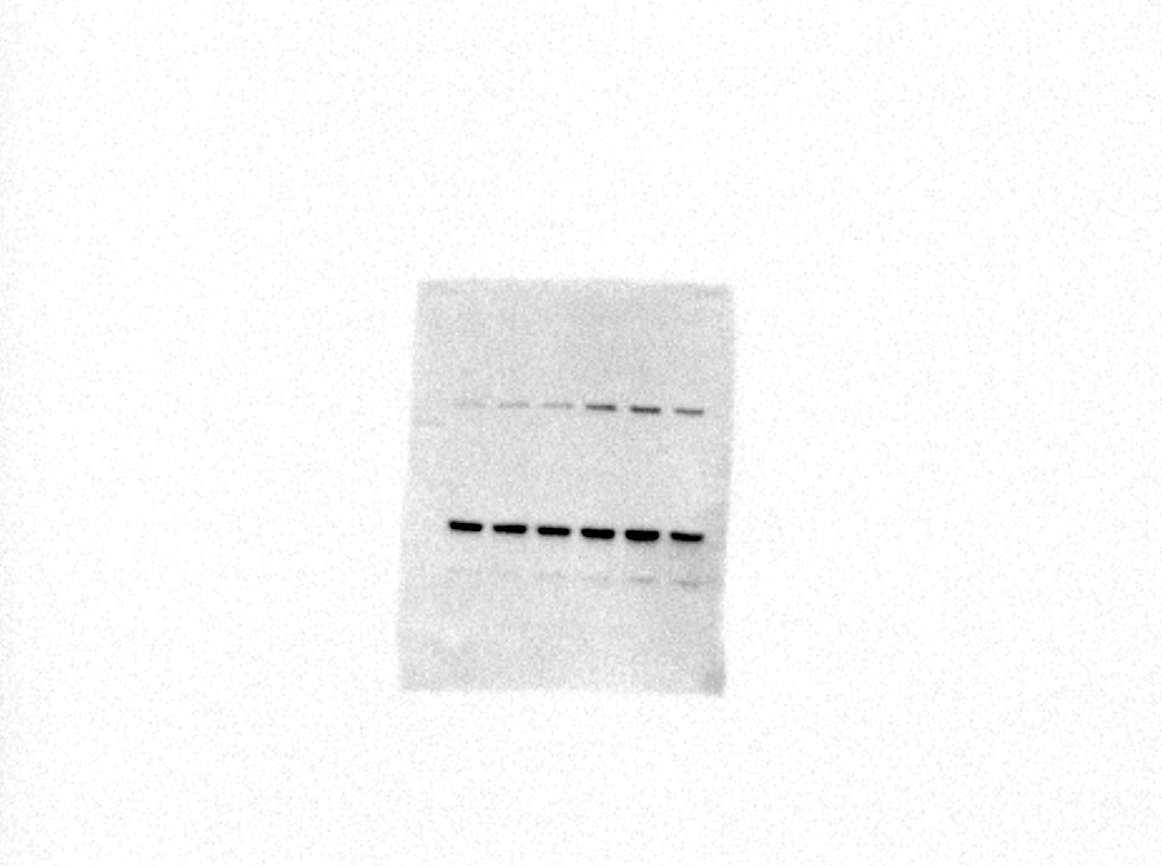


MEST-Placenta


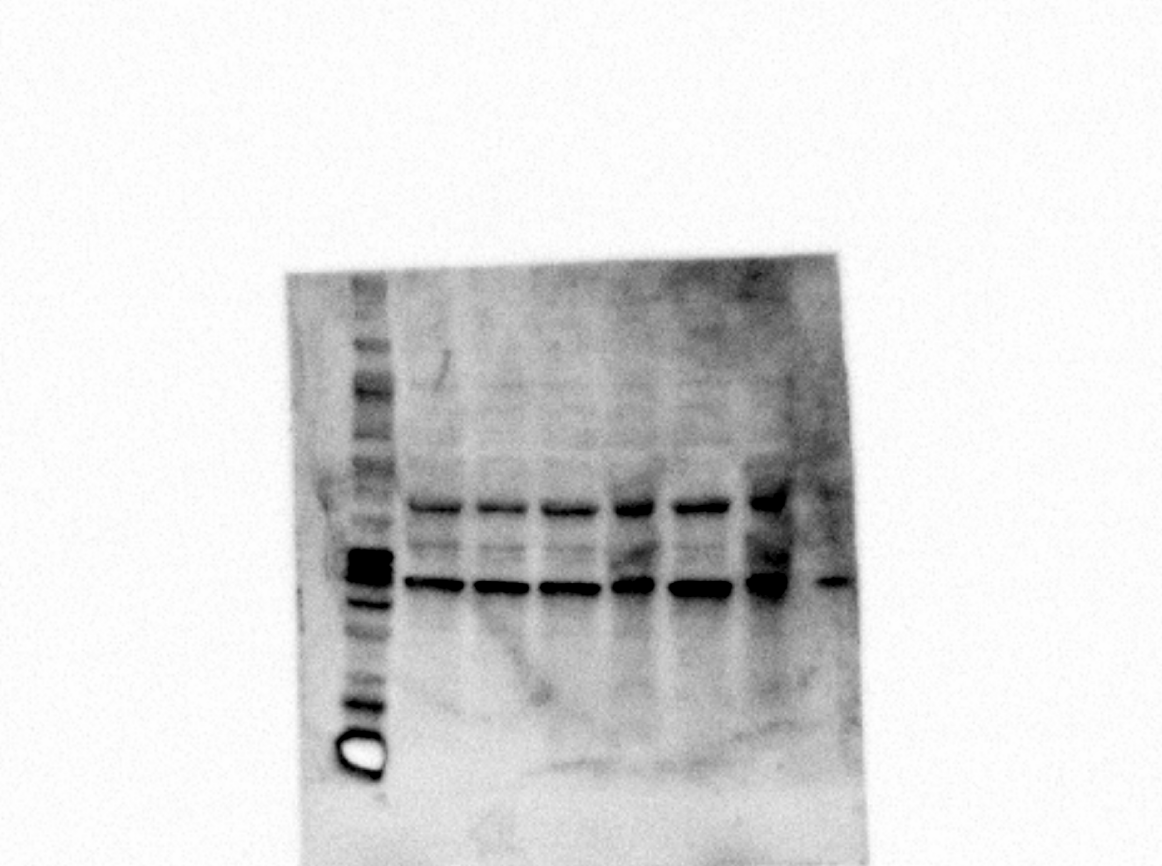


COIP-ESR1 -TET2


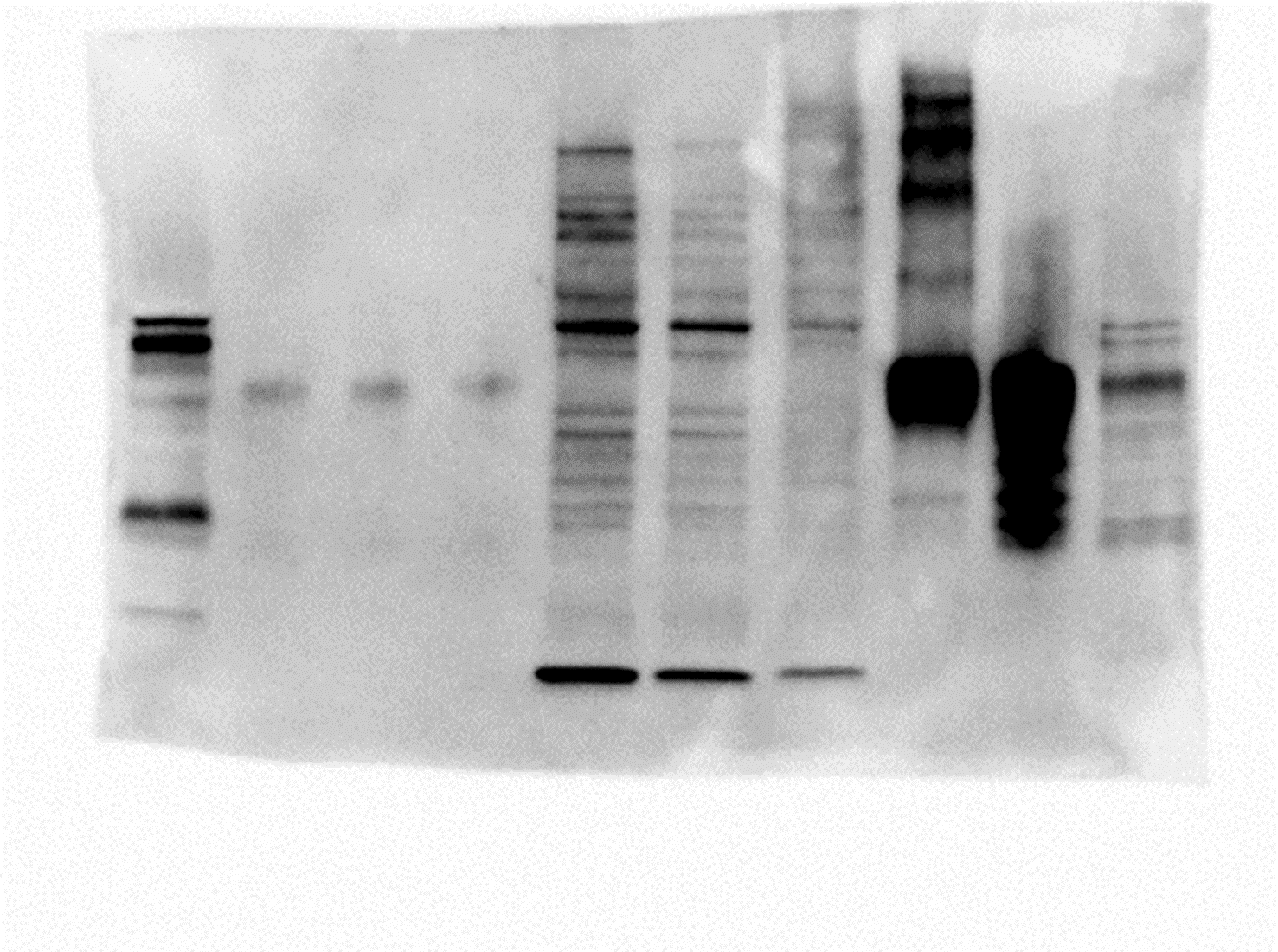


CHIP IMAGE-MEST


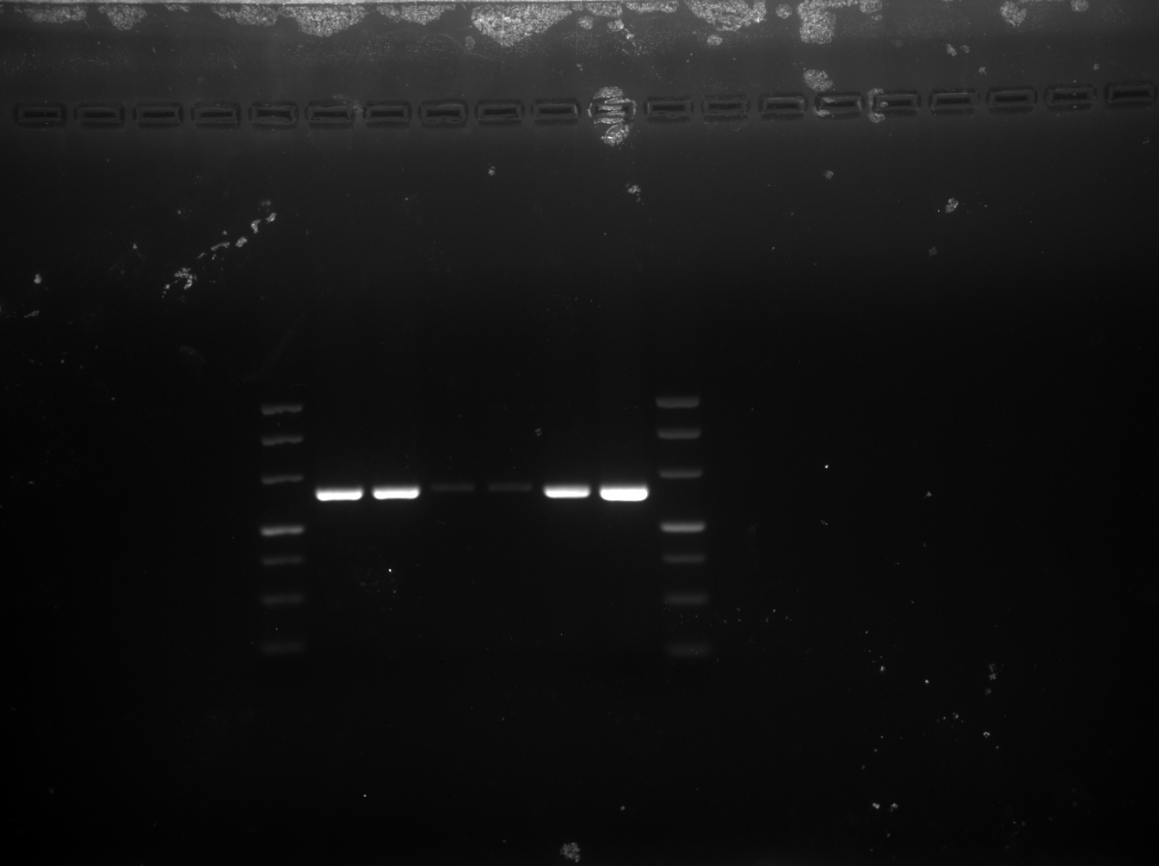

Supplement: Supplementary file 11 — Additional file 11. [file 12964_2024_1516_MOESM11_ESM.docx]
